# Model supports asymmetric regulation across the intercellular junction for collective cell polarization

**DOI:** 10.1371/journal.pcbi.1012216

**Published:** 2024-12-17

**Authors:** Katherine Levandosky, Calina Copos

**Affiliations:** 1 Department of Mathematics, Northeastern University, Boston, Massachusetts, United States of America; 2 Department of Biology, Northeastern University, Boston, Massachusetts, United States of America; Lehigh University, UNITED STATES OF AMERICA

## Abstract

Symmetry breaking, which is ubiquitous in biological cells, functionally enables directed cell movement and organized embryogenesis. Prior to movement, cells break symmetry to form a well-defined cell front and rear in a process called polarization. In developing and regenerating tissues, collective cell movement requires the coordination of the polarity of the migration machineries of neighboring cells. Though several works shed light on the molecular basis of polarity, fewer studies have focused on the regulation across the cell-cell junction required for collective polarization, thus limiting our ability to connect tissue-level dynamics to subcellular interactions. Here, we investigated how polarity signals are communicated from one cell to its neighbor to ensure coordinated front-to-rear symmetry breaking with the same orientation across the group. In a theoretical setting, we systematically searched a variety of intercellular interactions and identified that co-alignment arrangement of the polarity axes in groups of two and four cells can only be achieved with strong asymmetric regulation of Rho GTPases or enhanced assembly of complementary F-actin structures across the junction. Our results held if we further assumed the presence of an external stimulus, intrinsic cell-to-cell variability, or larger groups. The results underline the potential of using quantitative models to probe the molecular interactions required for macroscopic biological phenomena. Lastly, we posit that asymmetric regulation is achieved through junction proteins and predict that in the absence of cytoplasmic tails of such linker proteins, the likeliness of doublet co-polarity is greatly diminished.

## Author summary

During collective movement, individual cells typically engage their autonomous polarity machinery, while being connected to their neighbors through adhesive cell-cell interactions. Despite advances in revealing the cell-cell interactions required for collective cell migration, a comprehensive picture of the molecular basis of intercellular communication for collective guidance is missing. To address this question, we devise a generalized mechanochemical model for cell polarity in a doublet and investigate how polarity signals are transmitted from one cell to another across seemingly symmetrical junctions. We have chosen to screen through all possible simple intercellular conditions of the Rho GTPase signaling circuit and/or F-actin structural dynamics. Our systematic approach provides information on over 300 distinct conditions and reveals the intercellular regulation provided by junctional proteins. In addition to predicting that only asymmetric interactions favor co-polarization, ensuring movement of the group in the same direction, our analysis also highlights the need for additional regulatory mechanisms for larger cell groups in geometrically non-constrained environments.

## Introduction

From bacterial to mammalian cells, cell polarity is essential in a multitude of functional contexts, including cell migration, division and differentiation, and development [1–5]. Cell polarity is manifested in molecular and morphological asymmetries across the cell [6, 7]. One fundamental question related to cell polarity is how an initially symmetric cell can spontaneously establish a polarized state, with a well-defined cell front and rear, but also show sensitivity to external guiding cues [8]. Cells are also known to engage in collective migration, which necessarily requires negotiation of the individual cell’s direction of movement with its neighbors across symmetric cell-cell junctions. Previous studies have shown that vectorial signaling requires mechanical coupling between cells through cadherin dependent cell-cell junctions [9–12]. This raises a second fundamental question: What are the underlying biochemical and/or structural interactions at cell-cell junctions that support co-orientation of polarity axes such that all cells in a group polarize in the same direction?

**Fig 1:**
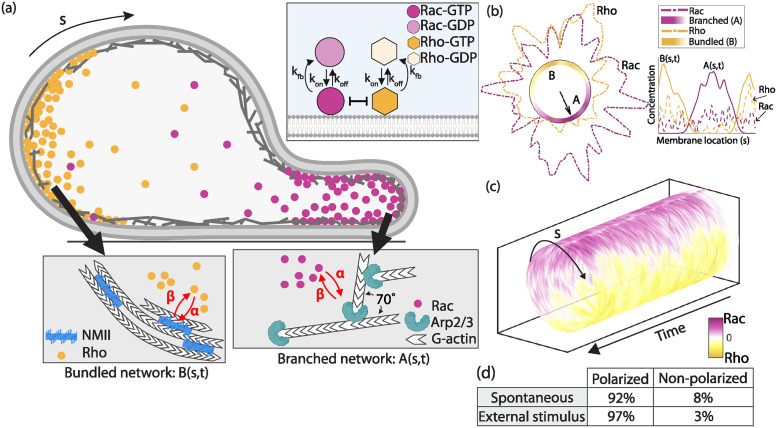
Model for spontaneous polarity in individual cells. (a) Side view schematic of front-rear polarity markers in a cell. Top inset: GTP-GDP cycling dynamics of Rho GTPases in the model. Bottom insets: Local, bidirectional crosstalk (red arrows) between Rho and actomyosin bundles (left) and Rac and Arp2/3 branched F-actin networks (right) ensures the simultaneous spatial organization of two distinct F-actin networks supporting the formation of a cell rear and front, respectively. (b) The outcome of one model realization shown with two representations: along circular boundary and along periodic 1D domain (inset). In the circular representation, heatmap plots of the branched (magenta) and bundled (yellow) F-actin networks are shown inside the cell. The GTPase concentrations, Rac (magenta) and Rho (yellow), are plotted outside the cell membrane. A front-rear polarity axis is drawn with a black arrow. Inset: same simulation output along the 1D periodic domain with continuous lines for F-actin structures and dashed lines for Rac/Rho molecules. (c) Rac and Rho concentrations in space and time averaged over 100 independent realizations—a cylinder slice corresponds to the concentrations at one fixed time point. (d) Summary of polarization probability for 100 realizations.

The first question is well studied, both conceptually with theoretical approaches reviewed in [13, 14], and experimentally, by characterizing signaling pathways [15, 16]. The polarization of an initially non-polarized cell is a symmetry breaking phenomenon: in the case of essentially isotropic cells, the continuous angular symmetry is broken by polarization, which can happen spontaneously [8, 17], but is often controlled by upstream guiding cues [13], and noise can play an important role [18]. Polarity establishment arises primarily through the localization of specific proteins and lipids in the cell to specific regions of the plasma membrane, and often precedes motility [6, 7]. While the detailed molecular mechanisms differ between organisms, they involved a relatively small, conserved set of proteins—here, we focus on the Rho molecular circuit [3, 19] and specifically the GTP-GDP cycling of small GTPases Rac1, which promotes lamellipodial protrusions at the migrating front, and RhoA, which promotes contractility at the rear, (Fig 1a)—these proteins will be referred to as Rac and Rho, respectively, henceforth.

Cell polarization can also be associated with the rearrangement of the actin cytoskeleton, in which branched actin filaments form at the cell front while actomyosin contractile bundles segregate to the cell rear [4, 20, 21] (Fig 1a). Just as diffusible chemical activators and inhibitors trigger biochemical instabilities, structural instabilities can arise due to stochastic fluctuations in actin filament densities or mechanical feedback between motor proteins and cytoskeleton elements [22]. In structurally driven polarity systems, cells polarize due to the mechanical forces and the actin flow generated by these forces [4, 23, 24]. Two classic cases involving cytoskeleton-driven polarization are the formation of actin comet tails by intracellular pathogens [25] and the directional locomotion of keratocytes [4, 17, 26]. In both cases, the mechanical properties of the actin cytoskeletal network appear sufficient for polarization, which can be triggered by stochastic or induced asymmetries in the network. Although cell polarity can emerge from systems that are either chemical or mechanical, in many cases cell polarity depends on the interplay between the two to robustly break symmetry to initiate locomotion [27–31].

In collective migration, each cell individually contributes to the group’s migration by first breaking symmetry and establishing a polarity axis while maintaining physical contact with neighboring cells. For the group to move together in the same direction, further mechanisms are required for coordination of the polarity of their autonomous migratory machineries. Experimental work has focused on uncovering the links between cell signaling pathways and collective cell movement. In epithelial layer sheets, ERK signaling waves are tightly connected to mechanical forces to ensure collective migration [32]. In collectively migrating human umbilical vein endothelial cells, physical membrane protrusions termed ‘cadherin fingers’ interconnect the rear of leading cells to the front of follower cells [33]. These VE-cadherin rich structures are deeply connected to the actin cytoskeleton of both follower and leader cells and are thought to regulate Arp2/3 actin polymerization. Another clue into the intercellular coordination of the polarity pathway for collective migration comes from chemotaxing neural crest cells [34]. As neural crest cells ‘chase’ placodal cells, before cell-cell contact, neural crest cells have high, localized Rac activity at the cell front, but after contact, junction proteins (N-cadherins) inhibit Rac localization. Importantly, in cell ‘trains’, as exemplified by the migration of neural crest cells, collision and contact inhibition of locomotion (CIL) have been demonstrated to play a role *in vivo* by maintaining coherent directional migration of groups of cells [35]. A number of theoretical models have been developed to study the emergence of directed collective migration, reviewed in [36]. In particular, one model has focused on identifying the mechanisms, chemical and/or mechanical, that can account for CIL in interacting cell groups in confinement [37, 38]. Despite these combined efforts, the driving mechanisms to ensure coordination of collective symmetry breaking prior to migration remain elusive.

Here, rather than cells being pulled or pushed along, we systematically searched the intercellular biochemical and/or structural conditions for neighboring cells to coordinate their symmetry breaking processes ahead of movement. Specifically, we identified the simplest conditions at the cell-cell junction that ensure individual polarity axes are co-aligned towards a common direction across the cell group. We used a previously developed mechanochemical model for polarization of an individual cell [39]. The model was extended to a pair of cells and a number of interactions at the cell-cell junction are evaluated, including interactions which rely on the biochemical circuit, the structural circuit, or both.

Our results identified a very small set of interactions—asymmetric—of polarity markers which favor co-alignment or supracellular arrangement of front-rear axes in the doublet. Surprisingly, our finding held even if we assumed intrinsic cell-to-cell variability or an external signal orienting polarization rather than spontaneous polarization. We posit that these types of intercellular couplings at symmetric cell-cell junctions arise from ‘interpretation’ of mechanical forces by adhesion junctional proteins, which asymmetrically regulate the Rho signaling pathway in neighboring cells. In addition, we used our model to study collective polarization for larger groups of cells. One would expect that groups of 4 cells behave similarly to doublets, but, surprisingly, initial geometric arrangement also played an important role. We found that groups of 4 cells in a square (over single-file/chain) arrangement exhibited a wider variety of behaviors, ranging from co-alignment to clockwise or counterclockwise rotational alignment. We propose that this can be understood as due to the larger number of degrees of freedom, almost identical to behavior of cells in confined environments rather than flat surfaces [40]. Our findings suggest that additional regulatory mechanisms, perhaps CIL, are at play to sustain co-alignment organization of polarity axes in tissues.

## Model

### Molecular ingredients of the cell polarity model

Each cell in the doublet is capable of symmetry breaking and thus, establishing a front-rear axis through a generic mechanochemical polarity mechanism [39]. In a modeled cell, the geometry is a static circular one-dimensional periodic domain which represents the plasma membrane and a thin volume of cytoplasm adjacent to the membrane (Fig 1a). Within an individual cell, the location of the 4 front-rear polarity markers: two Rho GTPases (Rac and Rho, top inset Fig 1a) and two cytoskeletal networks (branched and bundled F-actin, bottom insets Fig 1a), is tracked along the arclength *s* at a given time *t*; therefore, the simulation captures the spatiotemporal evolution of these 4 polarity markers (Fig 1b). The model assumes a biochemical signaling circuit, based on small Rho GTPase active-inactive cycling, with positive, local, bidirectional feedback into an F-actin network circuit, based on ‘frontness’/‘backness’ cytoskeletal dynamics (Fig 1a). Alone, neither circuit can ensure front-rear symmetry breaking [39], but their coupling leads to robust spontaneous polarization in an individual cell as well as in the presence of an external stimulus (Fig 1b–1d). Briefly, we outline the dynamics assumed in each sub-circuit of the front-rear symmetry breaking model, but further details in S1 Text and model parameters in Table A in S1 Text.

The biochemical signaling circuit is based on the well-studied GTP-GDP cycling of the small GTPases Rac and Rho. In the model, each GTPase molecule cycles between two states: an active GTP-bound form, bound to the plasma membrane, and an inactive GDP-bound form, freely diffusing in the cytosol with diffusion coefficient *D*. The active molecule can unbind (dissociate) from the plasma membrane with rate *k*_off_, while an inactive molecule can bind (associate) with rate *k*_on_. Once bound, the molecule induces a positive feedback activation through recruitment of inactive molecules at rate *k*_fb_ to nearby locations on the plasma membrane. Rac and Rho molecules engage in mutual inhibition by blocking activation or recruitment events of opposite type molecules to nearby locations on the membrane [41–44]. To capture these kinetics, we use a stochastic formulation to track the position and the location of the activated, membrane-associated Rho GTPases at a given time.

For the structural circuit, we model the re-arrangement of the F-actin structures as a set of coupled reaction-diffusion equations, which describe the densities of branched protrusive actin network, *A*(*s*, *t*), and contractile bundled actomyosin network, *B*(*s*, *t*):

$$\frac{\partial A}{\partial t}=A(1+\alpha n_{\text{Rac}})-A^{2}-m_{0}\;AB+D\Delta A,$$ (1)

$$\frac{\partial B}{\partial t}=B(1+\alpha n_{\text{Rho}})-B^{2}-m_{0}\;AB+D\Delta B.$$ (2)

In addition to the free diffusion (with diffusion coefficient *D*) of the networks [17], we assume that the rate of growth of each network is proportional to its concentration but limited due to finite molecular resources (e.g. branching complexes, myosin II motors, etc.) [45]. A second reaction term is introduced to account for the competition (of strength *m*_0_) stemming from either mechanics or limited availability of molecular resources [17]. The coupling of the biochemical to the structural circuit is captured by the *α* term. The coupling assumes that the branched (bundled) network growth rate depends on local concentration of membrane-bound Rac (Rho) molecules. We note that the reverse direction of the coupling is also considered; it is incorporated by modifying the binding affinities (*k*_on_) of small GTPases such that they are not fixed rates but depend on the local concentration of each respective actin network. The quantitative mechanism suggested by the coupled model is simple: branched (bundled) actin networks support recruitment of Rac (Rho) molecules to the membrane, so Rac (Rho) molecules tend to segregate into separate parts of the cell. In turn, neither network can invade the other’s spatial domain, because Rac (Rho) molecules engage the branched (bundled) network.

### The cell-cell junction

**Fig 2:**
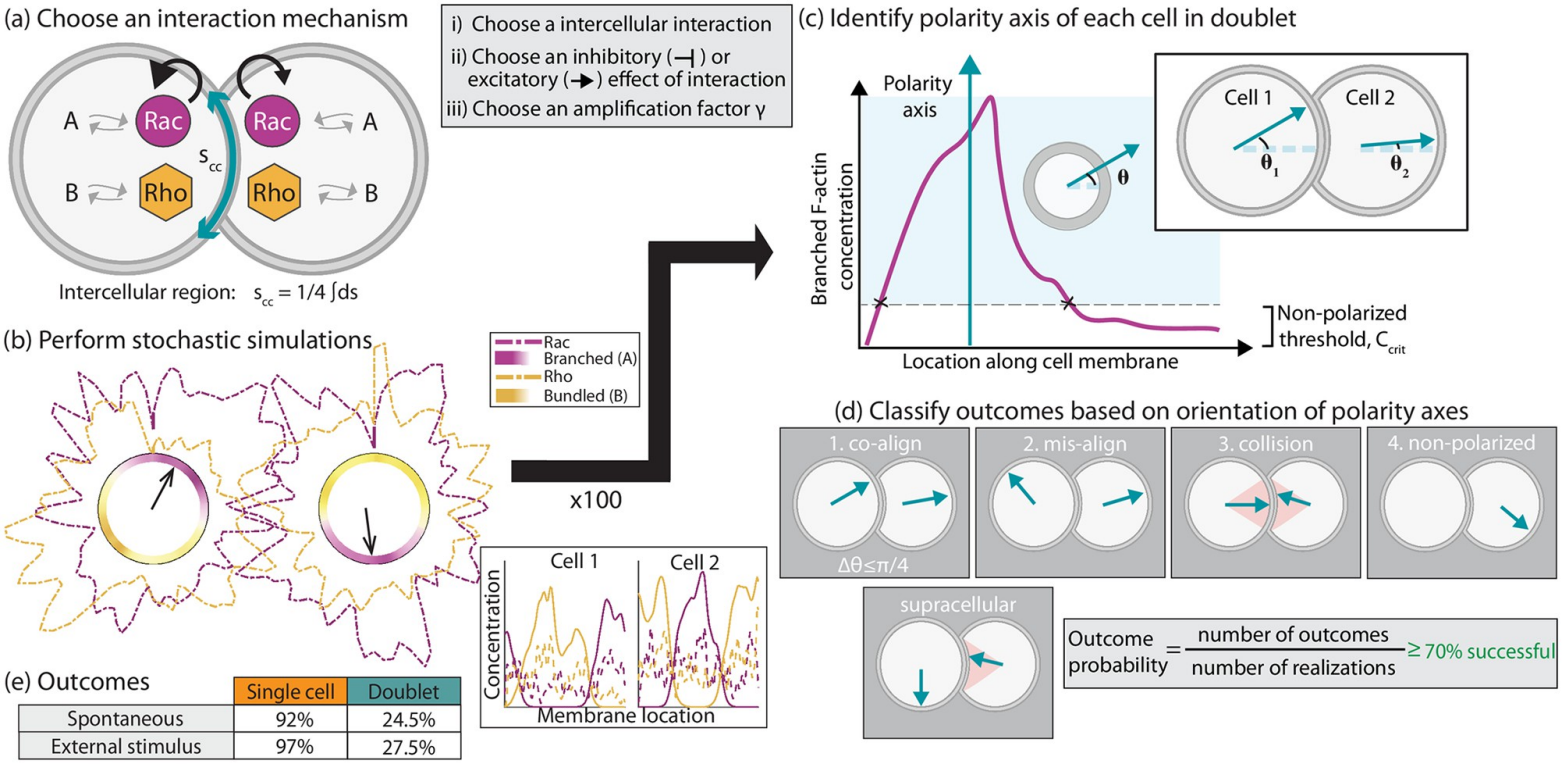
Schematic representation of workflow in a cell doublet. (a) A possible intercellular interaction in a pair of cells is selected. The interaction, which takes place at the intercellular region on the circular domain (cyan), can be based on Rho GTPases kinetics (black arrows), assembly of F-actin networks, or both. Inset: steps of choosing an interaction mechanism. (b) Sample simulation which results in misalignment orientation of polarity axes. Heatmap plots of the branched and bundled F-actin networks are shown inside the cell, while the Rac/Rho concentrations are plotted outside the cell membrane. The black arrows mark front-rear axes. Inset: Concentration of all 4 polarity markers along 1D domain (refer to Fig 1b for labels). (c) A front-rear axis is identified from the cell center to the midpoint of the region where the branched F-actin network is above a threshold value, *C*_crit_. Inset: the angle opening from the horizontal axis to the polarity axis is calculated for each cell. (d) Based on the orientation of the polarity axes in the doublet, an outcome is assignment. Possible outcomes are (1) co-alignment, (2) misalignment, (3) collision, (4) non-polarized. Supracellular arrangement overlaps co-alignment and misalignment outcomes. (e) Summary of probability outcomes for singlets (number of polarized cells) and doublets (number of doublets with both cells polarized in co-alignment arrangement) out of 100 model realizations.

We assume the doublet cells are equivalent (non-distinguishable), in the sense that they have the same biochemical kinetic rates and actin network parameters in the polarity model. Each cell establishes its own front-rear polarity axis, prior to migration. The pair maintains a static cell-cell junction, fixed to be 25% of the perimeter of each plasma membrane for all simulations (*s*_cc_, Fig 2a). To probe the effect of junctional protein complexes on regulating Rho GTPase signaling and/or F-actin network assembly, we assume that the junctional proteins affect the dynamics of the polarity markers.

To regulate the biochemical circuit, the binding (*k*_on_) and/or unbinding (*k*_off_) kinetic rates of the Rho GTPases are multiplied by an amplification factor (*γ*); the amplification factor is not one only at the intercellular region (*s*_cc_). Since binding and unbinding effects are considered separately, the amplification factor is only greater than or equal to one (*γ* ≥ 1).

The intercellular interaction of the F-actin structures similarly involves the reaction rates in Eqs (1) and (2); namely, the growth rates of each F-actin network can either be up-regulated or down-regulated, independent or dependent on the concentration of either actin network in the neighbor cell. As an example, we show the modifications in one cell, cell 1, but the same idea applies to its pair. To enforce this regulation of branched (*A*) or bundled (*B*) F-actin structures, the equations for the structural circuit in cell 1 were modified to

$$\begin{array}{ccc} \frac{\partial A}{dt} & = & A(1+\alpha n_{\text{Rac}}+\epsilon_{A})-A^{2}-m_{0}AB+D\Delta A, \end{array}$$ (3)

$$\begin{array}{ccc} \frac{\partial B}{dt} & = & B\left(1+\alpha n_{\text{Rho}}+\epsilon_{B}\right)-B^{2}-m_{0}AB+D\Delta B. \end{array}$$ (4)

The newly introduced rates in cell 1, *ϵ*_*A*_ and *ϵ*_*B*_, can either be constant:

$$\begin{array}{ccc} \epsilon_{A},\epsilon_{B} & = & \text{constant}, \end{array}$$ (5)

or dependent on the local concentration of F-actin networks in its neighbor, cell 2,

$$\begin{array}{ccc} \epsilon_{A} & = & \epsilon_{AA}A^{\left(\text{cell}\;\text{2}\right)}+\epsilon_{AB}B^{\left(\text{cell}\;\text{2}\right)},\;\;\epsilon_{B}=\epsilon_{BA}A^{\left(\text{cell}\;\text{2}\right)}+\epsilon_{BB}B^{\left(\text{cell}\;\text{2}\right)}. \end{array}$$ (6)

The rates, *ϵ*_*A*_ and *ϵ*_*B*_, are nonzero only on the intercellular region.

### Outcome classification

At each time point in the simulation, and for each cell, a front-rear polarity axis is calculated from the cell centroid to the point on the plasma membrane that corresponds to the midpoint of branched F-actin network (above a threshold level of *C*_crit_, Fig 2b and 2c). To determine if the pair co-oriented their polarity axes, the orientation and angle difference between polarity axes are determined. We identified a total of four possible distinct scenarios of the arrangement of the polarity axes (Fig 2d). The possible outcomes are:

**Co-alignment**: Polarity axes are roughly parallel to each other, with an angle difference less than 45 degrees (S1 Movie);
**Collision**: Both polarity axes point towards the cell-cell junction; the axes are roughly antiparallel (parallel vectors with opposite directions) and point within a 36-degree angle opening about the horizontal line (orange sectional area in (3.) in Fig 2d);
**Misalignment**: Neither one of the above cases, meaning that both cells polarize, but their polarity axes are neither in co-alignment nor collision arrangement, as defined above (S2 Movie);
**Non-polarized**: Either one cell or both cells fail to polarize; this can happen if either one of the networks never goes above threshold level *C*_crit_.

Given the stochastic nature of the Rac/Rho kinetics, 100 realizations are considered, and a probability outcome is computed as a proportion of the number of realizations in a particular front-rear axes arrangement.

Lastly, previous work has reported on the supracellular organization of motile groups of cells [46–49]. In our model, a supracellular (or leader-follower) arrangement is identified when the prospective leader’s polarity axis is aligned in any direction, but away from the cell-cell junction defined as a 45-degree contact region. Meanwhile, the prospective follower’s polarity axis is oriented toward the leader’s center-of-mass, within a 45-degree angle opening about the horizontal line (Fig 2d, S3 Movie). This configuration does overlap with co-alignment and misalignment arrangements.

## Results

### The absence of intercellular interactions produces sporadic co-alignment of front-rear axes in doublets

In the absence of interactions between cells, meaning that the kinetic rates and/or F-actin structural parameters are not changed at the intercellular junction, there was approximately a 25% chance for the pair to co-align their front-rear axes and thus, polarize in the same direction (Fig 2e). Even if we accounted for intrinsic variability in the kinetic rates and parameters of either biochemical or structural signaling modules across cells, the co-alignment outcome did not improve (first seven rows in Table 1). The co-alignment outcome also did not significantly improve for signal-induced polarization (Fig 2e)—in this scenario, we considered that only one of the two cells receives an external stimulus which is locally enforced by a spatial profile for the binding/unbinding rates for Rac molecules along the plasma membrane. Similar findings hold for the supracellular arrangement (Section D, Table B in S1 Text). These results inform us that for coordinated symmetry breaking across a pair of cells, the cell-cell junction must communicate the front-rear polarity signaling module, but which type of couplings (inhibitory or excitatory) of which signaling components (Rho GTPases and/or F-actin networks) can improve co-alignment of the polarity axes?

**Table 1:** A subset of molecular-based pathways of cell-cell coupling for a pair of cells based on experimental findings. The speculated couplings are implemented in the model and the outcome probabilities of co-alignment (Co-A.), collision (C.), misalignment (Mis.), or non-polarized (N.P.) arrangement are reported for 100 independent realizations of the model.

Pathway			Outcome Probability			
Cell 1	Cell 2	Refs.	Co-A.	C.	Mis.	N.P.
Uncoupled			0.25	0.03	0.57	0.16
Uncoupled with $10k_{\text{on}}^{\text{Rac}}$ in entire domain of cell 2			0.2	0.02	0.66	0.12
Uncoupled with $10k_{\text{on}}^{\text{Rho}}$ in entire domain of cell 2			0.18	0.03	0.63	0.16
Uncoupled with $10k_{\text{on}}^{\text{Rac},\text{Rho}}$ in entire domain of cell 2			0.21	0.03	0.68	0.08
Uncoupled with *ϵ*_*A*_ = 1 in entire domain of cell 2			0.22	0.02	0.69	0.07
Uncoupled with *ϵ*_*B*_ = 1 in entire domain of cell 2			0.2	0.03	0.63	0.14
Uncoupled with *ϵ*_*A*,*B*_ = 1 in entire domain of cell 2			0.18	0.04	0.78	0
Elevated Rho unbinding (*γ* = 1000)	Elevated Rho unbinding (*γ* = 1000)	[10, 50, 51]	0	0.64	0.15	0.21
Elevated Rho unbinding (*γ* = 1000, conc. dep.)	Elevated Rho unbinding (*γ* = 1000, conc. dep.)	[10, 50, 51]	0.02	0.37	0.5	0.11
Elevated Rho unbinding (*γ* = 1000)		[10, 50, 51]	0.24	0.13	0.48	0.15
Elevated Rac binding (*γ* = 1000)	Elevated Rac binding (*γ* = 10)	[12, 52, 53]	0.19	0.16	0.61	0.04
Elevated Rac binding (*γ* = 1000, conc. dep.)	Elevated Rac binding (*γ* = 1000, conc. dep.)	[12, 52, 53]	0	0.44	0.56	0
Elevated Rac binding (*γ* = 1000)		[12, 52, 53]	0.24	0.11	0.61	0.04
Up-regulated branched	Up-regulated branched	[54–57]	0	0.64	0.36	0
Branched promotes Rho and bundled promotes Rac		[58]	0.44	0.01	0.51	0.04
Mutual enhanced Rac/Rho antagonism		[59]	0.26	0.02	0.60	0.12
CIL		[37]	0.01	0	0.56	0.43
CIL and COA		[37]	0	0	0.67	0.33

### Speculated intercellular interactions for cell doublet polarity

We identified around a dozen speculated mechanisms that have been proposed based on biological experiments; to the best of our ability, we translated the experimental findings into specific local membrane affinities of one or more of the front-rear polarity components (Table 1 and Table B in S1 Text). The resulting outcome probabilities (out of 100 realizations for each interaction) are listed in the last columns of Table 1. This table represents only a small subset of a larger preliminary screening (of over 300 interactions) that was done as a first pass (refer to S4 for details). In our model, we found that many of the speculated interactions do not improve orientation in the same direction of the polarity axes of the doublet. We found that the majority of the tested interactions predominantly produced either collision or misalignment configurations (2 and 3 in Fig 2d). This is a likely outcome since, for example, increased Rac binding at the cell-cell junction in both cells will lead to the formation of protrusive fronts pointing towards the cell-cell junction due to the positive feedback between Rac and branched F-actin. Symmetric reciprocal unbinding leads to similar results—for example, increased Rac unbinding at the junction, predisposes Rho binding which will place Rac at the opposite side resulting in a protrusive cell front pointing away from the intercellular region in both cells, and thus high likelihood of misaligned arrangements (2 in Fig 2d). We also considered interactions where kinetic rates are not constant but concentration dependent, yet no reported significant differences in the outcomes (rows 9, 12, Table 1).

The lack of successful likeliness of co-alignment arrangement motivated us to pursue a second, more systematic screening. To reduce the computational complexity and exploit the bidirectional feedback between the structural and biochemical circuits, we performed two separate, exhaustive searches: one of the biochemical interactions and another of the structural, or F-actin network, interactions. This approach allowed us to identify simple motifs of intercellular interaction and score the outcomes based on likeliness to achieve co-alignment of front-rear axes in the doublet. In the biochemical circuit, we considered all possibilities of up-regulation in either binding or unbinding rates (*k*_on_, *k*_off_, respectively) for either Rac, Rho, or both, independently in each cell in the doublet. This included 4 parameters with 5 choices of the amplification factor (default, 10-fold, 100-fold, or 1000-fold increase) for a total of ∼ 100 interactions, minus repetitions. Next, in the structural circuit, similarly, we considered all possibilities for linear changes in growth rates of either branched, bundled, or both networks for a total of 162 pathways involving 4 parameters and 3 choices (default, decrease, increase). The counts in either search do not cover more complex schemes like concentration dependent rates or crosstalk between biochemical and structural circuits, which were additionally performed. What was not considered are nonlinear dependencies of the rates or other more complex interactions like multiple interacting components. We defined an interaction ‘successful’ if it resulted in over 70% likeliness for co-alignment arrangement (Fig 2d), as it represents roughly a three-fold increase over the uncoupled case [60].

### Asymmetric crosstalk of the biochemical signaling circuit significantly improves doublet co-orientation of polarity axes

**Fig 3:**
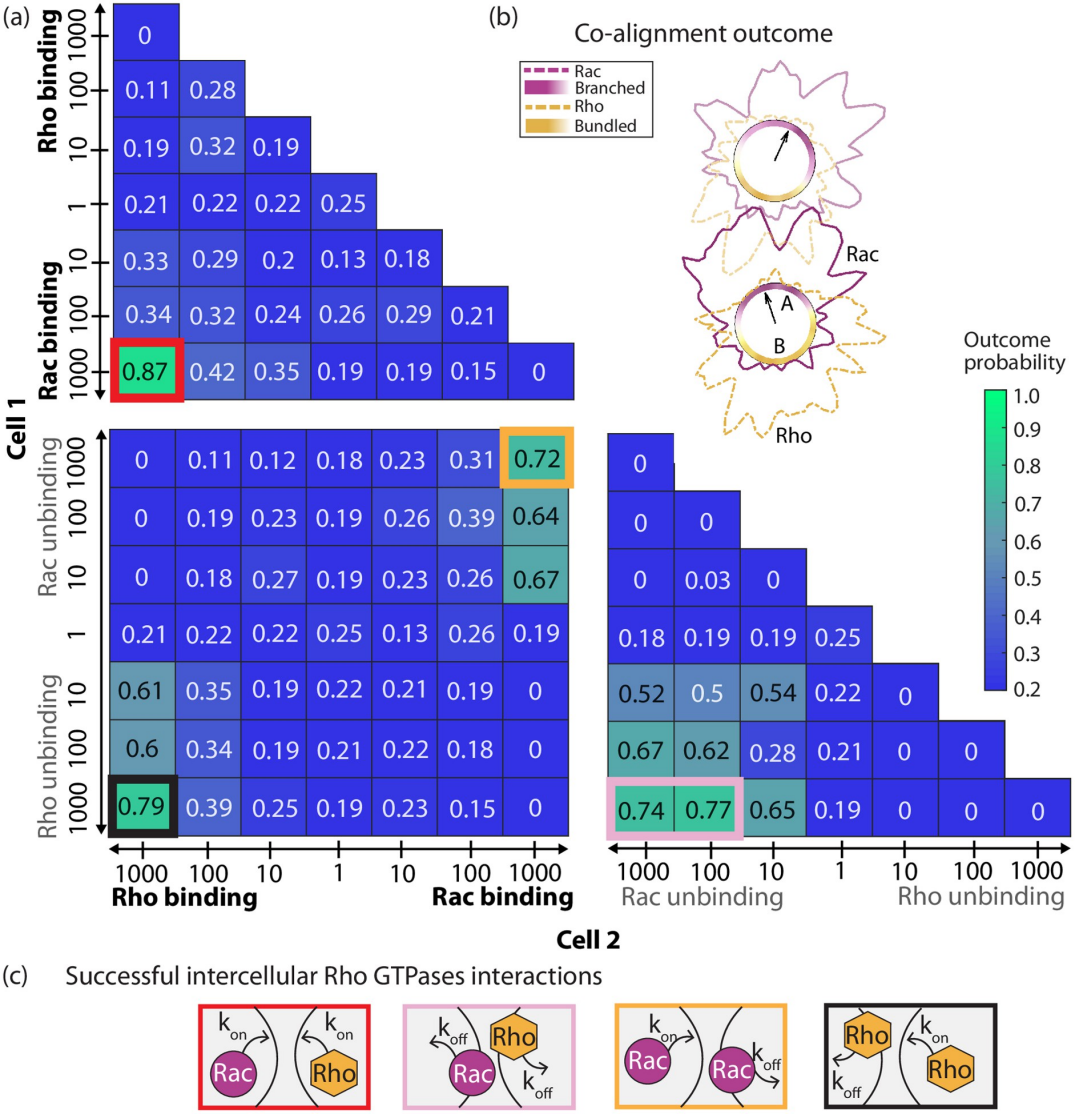
Probabilities of co-alignment arrangement under the assumption of intercellular regulation of Rho GTPase kinetics. (a) The co-alignment outcome probabilities for systematic combinations of amplified binding (*k*_on_, boldface) and/or unbinding (*k*_off_, gray) rates of Rac and/or Rho at the intercellular junction. The numerical value and box color represent the outcome probability for doublet co-alignment arrangement. The numbers along the axes indicate the amplification factor (*γ*) while the label indicates which rate, in which cell, was affected. Modifications done in cell 1 are shown along the *y*-axis, and cell 2 along the *x*-axis. The outlined boxes indicate successful interactions, and the color corresponds to the interaction motif in (c). (b) Sample doublet simulation resulting in co-alignment arrangement. Different opacity is used to distinguish between the cells. (c) Emergent successful intercellular pathways based on Rho GTPase signaling.

We asked what type of biochemical interactions of small GTPases at the cell-cell junction are needed to establish collective orientation of polarity axes in the cell doublets. The molecular origin of the interaction may involve direct molecular contacts between juxtaposed cells or indirect couplings mediated by diffusible molecules. Here, we abstracted the molecular details and assumed that either the binding (*k*_on_) or the unbinding (*k*_off_) kinetic rate of one GTPase is increased by an amplification factor (*γ*) at the cell-cell junction in one or both cells (Fig 3a). The *x*- and *y*-axes in Fig 3a indicate the value of the amplification factor in cell 1 and 2, respectively. The factor can either be constant (concentration independent) or proportion to the number of molecules in the neighboring cell (concentration dependent). First, we discuss concentration independent regulation. We found only one type of interaction is successful (70% or higher probability for co-alignment outcome): strong asymmetric regulation of the Rho GTPases (Fig 3b and 3c, S1 Movie). Asymmetric regulation across the cell-cell junction can happen through one of 4 ways: binding (or unbinding) of one Rho GTPase in one cell and similar action of binding (or unbinding) of the complementary Rho GTPase in neighboring cell (red and pink boxes), or complementary kinetics, binding in one cell and unbinding in neighboring cell, of the same molecule type (yellow and black boxes)). Irrespective of the type of asymmetric coupling, a probability of 70% or greater is attained for either co-alignment arrangement or supracellular arrangement (Fig A in S1 Text), but for supracellular arrangement, we found that the region of successful outcomes expands slightly to include smaller values of the amplification factor (white asterisks, panel (a) in Fig A in S1 Text).

Next, we explored whether collective polarization of doublets could be improved by up-regulation GTPase kinetics in a concentration dependent manner rather than constant. The reason is that a bound Rho GTPase could provide positive feedback across the tight junctions and adherens junction through paracellular diffusion of GAPs [61, 62]. This was implemented by multiplying the amplified kinetic rate coefficient by the amount of molecules in the neighboring cell engaged in that specific interaction pathway. For example, if the concentration independent intercellular interaction was increased binding affinity of complementary Rho GTPases (red box, Fig 3a), the concentration dependent amplification factors would be “$1000\;n_{\text{cell}\;\text{2}}^{\text{Rac}}$” for Rho binding rate in cell 1 and “$1000\;n_{\text{cell}\;\text{1}}^{\text{Rho}}$” for Rac binding rate in cell 2. *n* denotes the number of nearby active, membrane-bound molecules, of the type stated in the superscript, and in the cell indicated by the subscript. Surprisingly, we found a significant drop in the likeliness of co-alignment of doublets (bottom left, Fig B in S1 Text). The reason is that up-regulation of binding rates will be minimal if the corresponding molecule concentration is zero or low. In the case of supracellular arrangement, the results were qualitatively the same but notably asymmetric kinetics for Rac does not yield a high probability outcome (top right, Fig B in S1 Text). We concluded that, in our model, concentration independent strong asymmetric regulation of Rac/Rho is more likely to yield self-organization of front-rear axes in the same direction, prior to doublet migration.

### Co-alignment of front-rear axes can also be achieved through regulation of the F-actin structures

**Fig 4:**
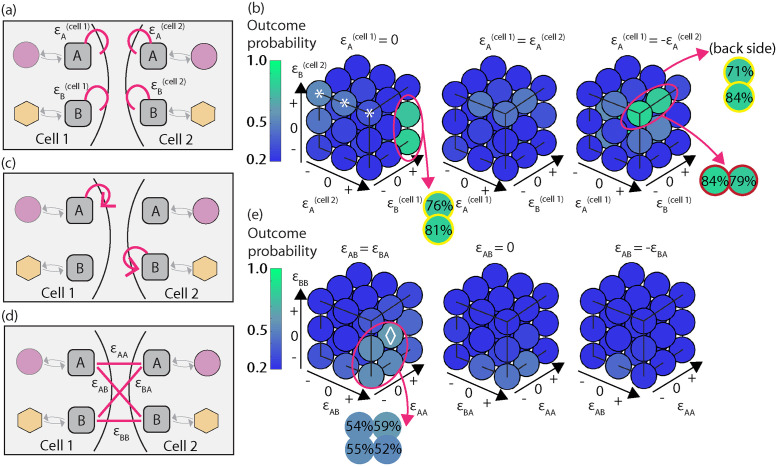
Probabilities of co-alignment arrangement with intercellular regulation of the assembly rates of branched and bundled F-actin networks. (a,d) Network interaction schematics for (a) concentration independent and (d) concentration dependent increase of growth rates of either branched (*A*) or bundled (*B*) networks. (b) Probabilities of co-alignment arrangement are projected onto a 3D parameter space exploration with the additive F-actin network growth rate constants taking on either positive, zero, or negative values in Eq (5). White asterisks indicate polarization probabilities for mutual excitation-inhibition of like F-actin networks. (c) Schematic of mutual excitation of complementary networks, which favors same-direction polarity in the doublet. (e) The same 3D parameter space exploration is used to show the probabilities co-alignment outcomes for concentration dependent network growth rate in Eq (6). White diamond denotes highest probability outcome.

After considering intercellular communication of GTPase circuits in two neighboring cells, we probed whether co-orientation of polarity axes can be established through only regulation of F-actin structures at the junctional region (Fig 4). The dynamics of the F-actin circuit were as initially described in the Model section (Eqs (1) and (2)), except at the cell-cell junction, where the growth rates of each F-actin network can be either up-regulated or down-regulated, independent or dependent on the concentration of F-actin in the neighboring cell (described in Model section). All possible network couplings, including diminishing (negative) and increasing (positive), were explored for a total of 162 pathways: 162 = 3^4^ (3 choices: promote, inhibit, none; 4 parameters: *ϵ*_*A*_, *ϵ*_*B*_ for both cells) + 3^4^ (3 choices: promote, inhibit, none; 4 parameters: *ϵ*_*AA*_, *ϵ*_*AB*_, *ϵ*_*BA*_, *ϵ*_*BB*_).

When network crosstalk was regulated in a concentration independent manner (Fig 4a), co-alignment arrangement was achieved with high likeliness in only 8 interactions (Fig 4b); all shared one common motif: reciprocal excitation of complementary F-actin structures. The motif requires that the up-regulation of the growth rate of one network type, and simultaneous up-regulation of the growth of the other network type, in the neighboring cell. To illustrate this, we considered the simultaneous increased growth rate of bundled network (*B*) in cell 1 but branched network (*A*) in cell 2 ($\epsilon_{B}^{\left(\text{cell}\;\text{1}\right)},\epsilon_{A}^{\left(\text{cell}\;\text{2}\right)}>0$), while the other two rates can take on non-positive values (yellow outline, Fig 4b). This scenario produced 4 cases with co-alignment likeliness ranging between 71 to 84%. The other 4 additional successful interactions emerged from the mirror case of up-regulation of branched network in cell 1 and bundled network in cell 2 (red outline, Fig 4b). We note that two of these interactions are not shown; they correspond to the case of zero increase in the growth rate of branched F-actin in cell 1. An expected interaction pathway motif was the mutual excitation-inhibition of the same type of F-actin structures; for example, increased growth rate of bundled actin in one cell but decreased growth rate (of the same network) in its neighbor. To our surprise, not all parameters within this interaction pathway produced high probability for either co-alignment of front-rear axes (white asterisks, Fig 4b) or supracellular arrangement (white asterisks, panel (a) in Fig C in S1 Text). The theme of our findings from crosstalk of the Rho signaling circuits expands to F-actin circuits—co-orientation of front-rear axes in the doublet can be achieved only through enhanced formation of complementary networks across the cell-cell junction, a ‘push-n-pull’-like mechanism (Fig 4c).

Results were remarkably different for concentration dependent interactions (Fig 4d). In this case, co-alignment outcome likeliness never reached 70% (Fig 4e), but did for supracellular arrangement (panel (b) in Fig C in S1 Text). In this leader-follower arrangement, the model predicted that the probability outcome is maximized for reciprocal (*ϵ*_*AB*_ = *ϵ*_*BA*_) and excitatory (*ϵ*_*AB*_, *ϵ*_*BA*_ > 0) couplings between branched and bundled networks in neighboring cells (inset, panel (b) in Fig C in S1 Text). Moreover, these successful interactions required like-networks to either engage in either no interaction or inhibition (*ϵ*_*AA*_, *ϵ*_*BB*_ ≤ 0) across the cell-cell region. In this scenario, the co-alignment arrangement was achieved in 52–59% of the cases (inset, Fig 4e; white diamond indicates largest value), but the likeliness of supracellular arrangement was higher, around 69–76%, with no collisions.

### Cell-to-cell variability in model parameters does not augment the set of intercellular interactions that favor collective polarity orientation

Can cell-to-cell variability in the polarity machinery, either externally induced or intrinsically generated, account for other regulatory mechanisms for co-orientation of polarity axes in the doublet? Specifically, would intercellular conditions in either the biochemical or F-actin circuit change when cell-to-cell variability is considered? To test this hypothesis, we assumed that one cell, cell 2, in the doublet has more responsive Rho GTPase activity by elevating the baseline affinity for Rac and/or Rho association rate (Table C in S1 Text) or greater baseline growth rate for the branched and/or bundled network (Table D in S1 Text). We scanned a subset of the possible interactions at the intercellular junction and quantified the polarization outcomes. The subset of probed cell-cell regulatory interactions were: the 4 asymmetric Rho GTPase interactions schematically illustrated in Fig 3c, up-regulation of Rho unbinding in one or both cells, up-regulation of Rac unbinding in both cells, enhanced small GTPase mutual antagonism, CIL and COA, and the F-actin network crosstalk (as in white diamond, Fig 4d; *ϵ*_*AB*_, *ϵ*_*BA*_ > 0 but *ϵ*_*AA*_ = *ϵ*_*BB*_ = 0).

In the case of more responsive GTPase activity, we assumed that one cell in the doublet has higher binding rate for either Rac or Rho or both GTPases—the rate was increased by a factor of 10 along the entire domain before additional assumptions for intercellular communication were made. With small differences, co-alignment (Table C in S1 Text) arrangement was favored if the intercellular interaction was one of the 4 asymmetric Rho GTPase crosstalk ways or F-actin structural crosstalk. Notably, the F-actin structural crosstalk interaction was not successful in identical doublet simulations (white diamond, Fig 4d). Next, we considered cell-to-cell variability with respect to the F-actin dynamics—in one cell, we assumed a higher network growth rate for either bundled, branched, or both actin networks. The model results for structural variability were nearly the same as in the case of biochemical cellular variability; co-alignment arrangement was a likely outcome if the intercellular interaction was one of the 4 asymmetric Rho GTPase crosstalk or F-actin network crosstalk (Table D in S1 Text). The results were similar for supracellular arrangement (Tables C and D in S1 Text). There was one notable exception—if one cell had higher baseline growth rate of bundled network, most of the asymmetric Rho GTPase or F-actin crosstalk ways did not lead to high probability of co-orientation of the polarity axes. In this case, the only successful intercellular communication required asymmetric Rho kinetics. In summary, in these interrogated pathways for cell-to-cell variability, we found that the same intercellular communication motifs, as in the case of identical cells, ensured co-orientation of front-rear axes of the doublet.

### The same set of intercellular interactions are favored for external stimulus-driven polarization in the doublet

**Fig 5:**
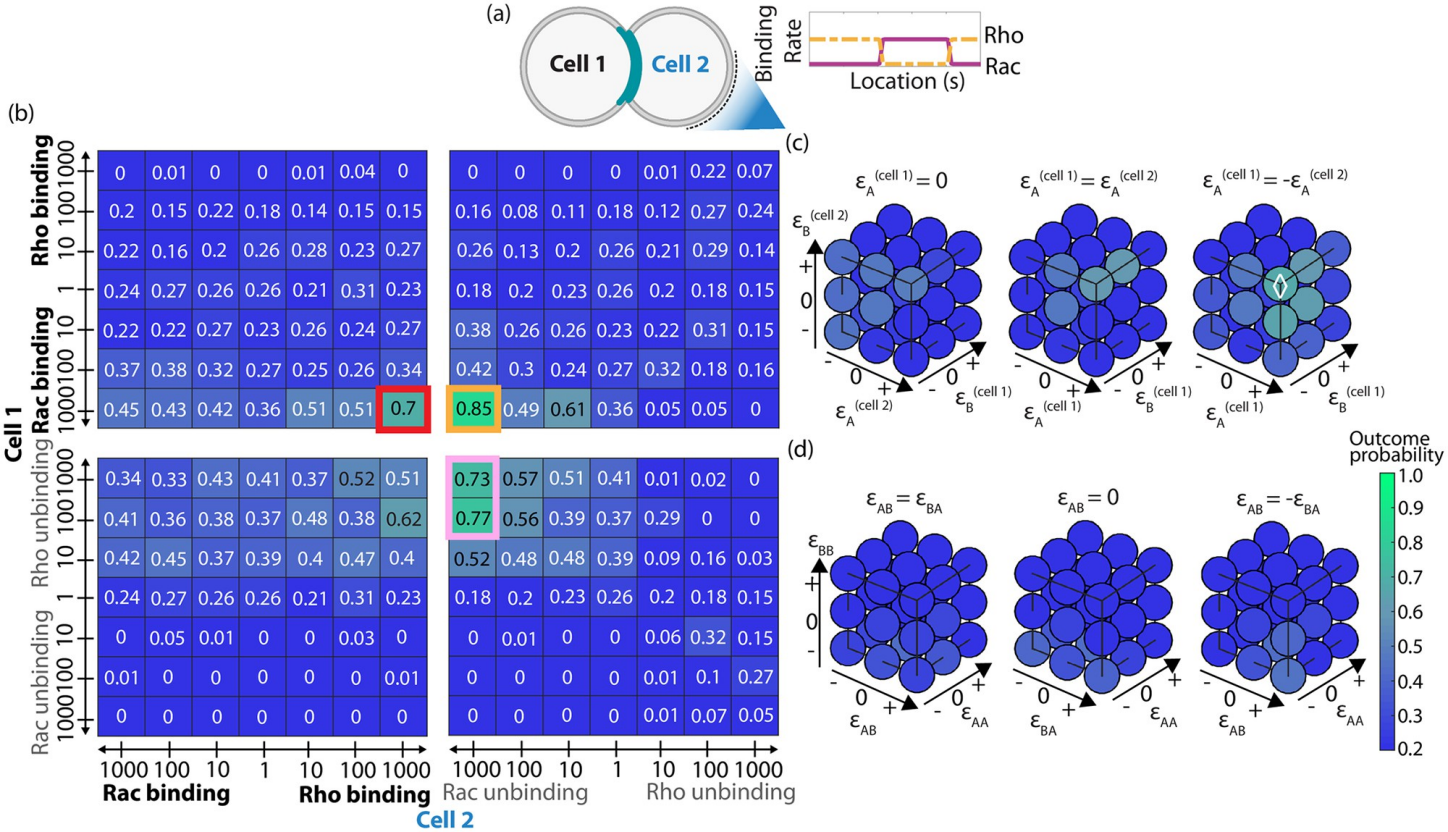
In the presence of an external stimulus applied to one cell in the doublet, the outcome probabilities of co-alignment arrangement are shown for intercellular crosstalk of either structural or biochemical circuits in the model. (a) An external stimulus (blue gradient) imposes a directional bias on the kinetic rates of both polarity proteins Rac and Rho. Inset: profile of kinetic rates for Rac binding (solid, magenta) and Rho binding (dashed, yellow) around the cell boundary. (b) Model outcome probabilities in the presence of an external stimulus applied to cell 2, with interactions of Rho GTPases as in Fig 3a. The outlined boxes indicate 70% or larger likeliness for the arrangement. The color of the outline matches the interaction schematic in Fig 3c. (c-d) Parameter space exploration projected onto 3D space for (c) additive network growth constants (Eq (5)), and (d) network-dependent growth rates (Eq (6)). White diamond denotes highest probability outcome.

To determine whether the doublet model exhibits sensitivity and adaptation to external signals, we simulated polarization in the presence of a directional bias. Trivially, in our model, if both cells received the same external signal, any intercellular coupling, including no coupling, resulted in both cells polarized in the direction of the signal [39]. Instead, only one of the paired cells was exposed to (and responds to) the external stimulus, and we probed what type of regulation of the polarity pathway at the cell-cell junction is needed to ensure that the nonexposed cell polarizes in the same direction as the stimulus-driven cell. To impose an external stimulus in one cell, we assumed that the binding rates for Rac/Rho molecules are non-constant along the plasma membrane, which is equivalent to a directional bias, as shown in Fig 5a—the Rac binding rate varied oppositely to the Rho binding rate, as the spatial complement of the curve: the sum of Rac and Rho binding rates was held fixed. The cell subject to an external stimulus was labeled as ‘cell 2’. As in [39], we report that in cell 2 a polarized state evolved from random initial conditions, with a Rac peak with the same orientation as the external bias (Fig 2e), but not necessarily in the neighboring cell. A search of intercellular pathways that could effectively communicate the signal across the intercellular junction was performed, and we found qualitative differences between spontaneous and stimulus-induced co-polarization of the cell doublet (Fig 5b–5d). One important difference was that any pathways based on structural interactions were unlikely to yield co-alignment (but did successfully give rise to supracellular arrangement) of front-rear axes in the doublet, detailed below.

#### Asymmetric regulation of Rho GTPases

In addition to the assumptions for GTPase kinetic rates in cell 2 due to the external stimulus, we enforced that neighboring cells engage in biochemical intercellular crosstalk through local up-regulation of binding and/or unbinding rates of either GTPase (Fig 5b, S4 Movie). Unlike the spontaneous case, there were more cases to be explored, since the symmetry of the doublet is lost (as cell 2 was subjected to an external stimulus). The same motif of asymmetric regulation of small GTPases across the common boundary emerged (outlined boxes, Fig 5b), albeit three of the four previously reported asymmetric interactions.

The three interactions that produced successful outcomes are: (1) up-regulation of binding rates and (2) unbinding rates of complementary GTPases, or (3) oppositely regulated kinetic rates (binding/unbinding) of Rac. Notably, the absent pathway was opposite regulation of Rho kinetic rates (binding/unbinding). This case resulted in only ∼50% likeliness of co-alignment of front-rear axes; reduced since this would cause two competing fronts for cell 2 (or the absence of a rear). On the other hand, for supracellular arrangement the high probability interactions were exactly the same four as those identified for spontaneous polarization (panel (a) in Fig D in S1 Text). However, there were even fewer constraints in these successful interactions, as demonstrated by the larger region covered by outlined boxes. The only constraint we found is that the Rho unbinding rate cannot be too high in cell 2, as that would lead to the loss of the cell rear. In summary, the biochemical-based intercellular pathways in signal-driven polarization of the doublet approximately fall under the same umbrella of interactions identified successful for spontaneous polarization.

#### Push-n-pull of F-actin networks

In the case of F-actin network interactions, we report the partial collapse of an intercellular interaction that was successful for spontaneous polarization. To demonstrate this result, in addition to the external stimulus assumption for the spatial profile of GTPase kinetic rates of cell 2, the cells in the doublet engaged in F-actin crosstalk in a concentration independent or dependent way precisely as in the spontaneous case. A parameter scan was done for all possible combinations of F-actin structure interactions (Fig 5c and 5d).

The highest probability for co-aligning the front-rear axes in the doublet was achieved with concentration independent altered network growth rates, and even then the outcome probability did not exceed 67% (white diamond, Fig 5c). The interaction is reciprocal excitation of branched, in one cell, and bundled network, in the other cell. Hence, in the presence of an external stimulus, mutual interaction of F-actin networks across the intercellular junction was not sufficient to produce co-orientation of polarity axes. However, supracellular arrangement did successfully emerge for a number of concentration independent interactions in which the growth rate of branched F-actin was elevated in the non-stimulated cell, while the growth rate of bundled F-actin was either not changed or down-regulated in the neighboring, exposed cell (panels (b)-(c) in Fig D in S1 Text).

#### Response to switch in the orientation of the external stimulus

Finally, we assessed whether a polarized doublet shows sensitivity to a new signal direction by re-polarizing in the new direction. We initiated the cell doublet and applied an external stimulus located in the lower right corner of cell 2 (centered around *θ* = 275°, (Fig 5a). After a period of time (*t* = 5 seconds), we removed the stimulus and placed a new stimulus in the upper left corner of cell 1 (*θ* = 135°). Only a small subset (6) of intercellular interactions were explored in order to determine if our model doublet can re-polarize with this dramatic switch in not just directionality, but also the identity of the stimulus exposed cell (Table E in S1 Text). Regardless of the interaction, in most model realizations we found that after the signal switch, the cells failed to repolarize as all polarity molecules dissociated from the plasma membrane (S5 Movie). This motivated a second implementation of the signal switch; if all Rac and/or Rho molecules unbound, a neutralization process was initiated, much like the initialization process. The neutralization process reset Rac/Rho molecules by randomly placing 10% of Rac/Rho molecules around the plasma membrane. After implementing the neutralization process, we found highest (but unsuccessful) probability of co-alignment arrangement for concentration dependent up-regulation of unbinding rates of complementary Rho GTPases at the cell-cell junction (Table E in S1 Text). The scenario produced 55% of pairs co-aligning in the direction of the signal, but 79% of doublets (both cells in the doublet) polarized in the new direction of the signal (S6 Movie). The difference between co-alignment arrangement and both cells pointing towards the signal just comes from the fact that the signal is ‘wider’ than the angle we require for co-alignment.

### Time to achieve a co-polarized state is not reduced compared to an individual cell

**Fig 6:**
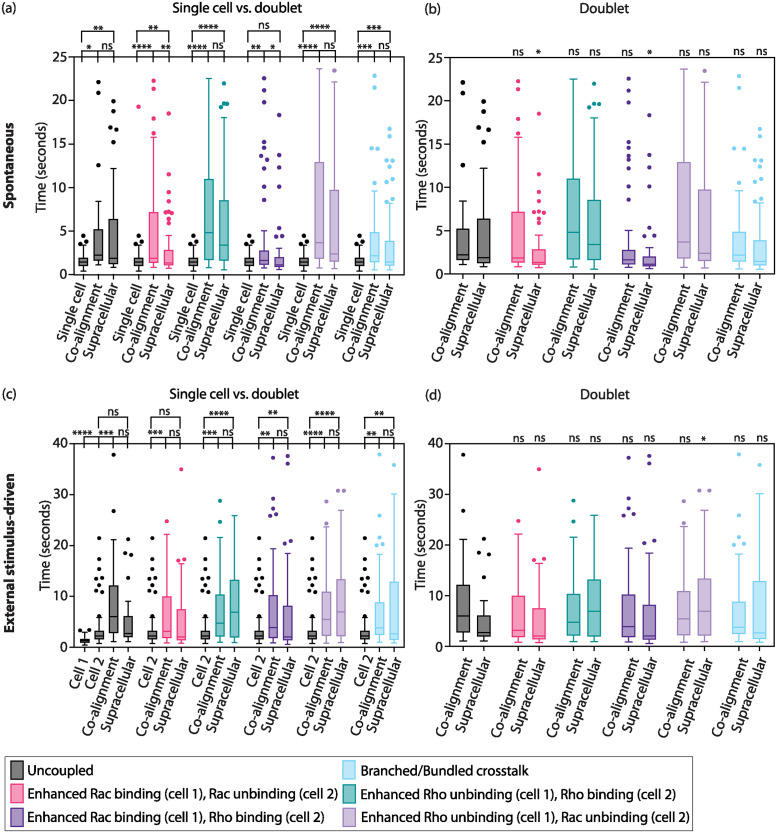
Time to co-polarize for a doublet is not reduced compared to that for an individual cell. (a, c) Comparison of time to reach a polarized state of single cell against doublet in the (a) absence or (c) presence of an external stimulus in either co-alignment or supracellular organization. (b, d) Comparison of time to reach polarized state of uncoupled doublet against doublet with an intercellular interaction in the (b) absence and (d) presence of any external stimulus presented to one cell only. Each color represents a particular interaction at the cell-cell junction region. The number of cases considered per interaction is 100. Welch’s ANOVA; n.s. p ≥ 0.05, *p < 0.05, **p < 0.01, ***p <0.001, ***p<0.0001. Boxes are the 25th to 75th percentiles, bars indicate ± interquartile range, and line denotes median value.

Next, we quantified the time to reach a polarized state for single cells and doublets with various intercellular interactions in order to determine whether a doublet can break symmetry more readily than an individual cell. A total of 5 intercellular interactions were considered, which include the 4 cases of asymmetric Rho GTPase regulation (Fig 3c), and the F-actin network mutual inhibition-excitation crosstalk (white diamond, Fig 4e). In our model, we found that the doublet always takes just as long or longer to break symmetry when compared to a single cell (Fig 6).

A polarized cell state is defined in Model section and reviewed here: both branched and bundled concentrations must be above the threshold level (*C*_crit_) and the orientation of the front-rear polarity axis is defined from the cell centroid to the midpoint of the threshold branched F-actin network concentration. Furthermore, to report the time to reach a polarized state, we ensured that the orientation of the axis remained fixed. This is especially relevant for doublets where the relative orientation of the polarity axes is important. Time to reach a fixed orientation of the polarity axis was defined to be the first instance when, within 100 time steps, the consecutive angle difference of the axis did not change more than a small amount (< *π*/12 radians).

For individual cells, the time for polarized state was longer with an external stimulus than without (spontaneous) (Fig 6a, gray). We attribute this outcome to the loss of bidirectional feedback between Rho GTPases and F-actin networks since, in the external stimulus scenario, the GTPases were no longer dependent on the F-actin network concentrations but rather had spatially fixed rates. For doublets, three sets of comparisons were performed; Fig 6a and 6c show comparisons of time to polarize of singlets against doublets and also time to polarize doublets in co-alignment against supracellular arrangement. Fig 6b and 6d show the comparison of the time to polarize uncoupled doublets against one of the 5 cell-cell couplings. First, we found that the time to reach a polarized state is as long or longer compared to an individual cell, indifferent of the presence or absence of an external stimulus. Further, that was true, indifferent of whether we looked for co-alignment or supracellular arrangement of polarity axes. Two couplings stood out as situations for which there was no statistically significant difference in the polarization time: asymmetric enhanced binding of complementary Rho GTPases (Fig 6a, indigo) for supracellular arrangement with no external signal and elevated binding/unbinding of Rac across the cell-cell junction (Fig 6b, pink) also for supracellular arrangement but with signal-induced polarization. Second, for a few cell-cell couplings, it was faster to achieve a polarized state in supracellular arrangement over co-alignment arrangement (Fig 6a, pink and indigo). Surprisingly, that was the case only for spontaneous polarization; in the case of stimulus-driven polarization, there was no difference in polarization time between the two polarity axes arrangements. Third, we found that intercellular couplings not only ensured higher likeliness of co-orientation of the cell group in the same direction but also could reduce the time to achieve a polarized state over the uncoupled scenario (Fig 6b and 6d).

### Geometric arrangement affects organization of polarity axes in larger groups

**Fig 7:**
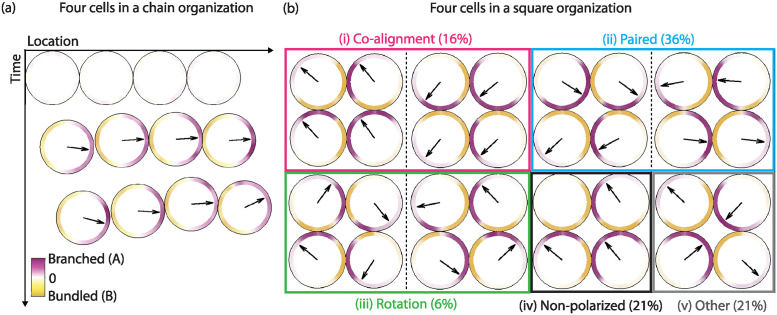
Polarization outcomes for 4 cells placed initially in two different geometric arrangements: Single-file (chain) or square. (a) Time evolution of a 4-cell cluster in a chain arrangement, where each cell in the cluster moves with a constant velocity in the direction of its polarity axis. (b) Possible outcomes of a 4-cell cluster in square arrangement. The probabilities are computed from 100 realizations of the quadruplet in a square configuration with F-actin network crosstalk implemented at all cell-cell junctions. With additional intercellular regions (lateral and transversal), a wider variety of arrangements of polarity axes emerges, including some suggestive of rotational motion.

Finally, we report on our findings for mechanisms for co-orientation of polarity axes for groups of 4 cells. As above, 5 intercellular interactions were considered, which included the 4 cases of asymmetric Rho GTPase regulation (Fig 3c) and the F-actin mutual inhibition-excitation crosstalk (white diamond, Fig 4e). Surprisingly, the successful co-alignment of front-rear axes also depended on the group’s prescribed geometric arrangement (Fig 7).

When cells were placed in a single-file arrangement (top Fig 7a, S7 Movie), we found very similar outcomes compared to cell doublets—either one of the 4 asymmetric interactions of Rho GTPases across the intercellular junction resulted in successful co-alignment of the doublets with probability ranging 85–96% (Table F in S1 Text). The reason why is straightforward: the cells (in a chain) can break symmetry in any direction, but once the cell-cell junction interactions were incorporated, this predisposes the cells to polarize in an axis perpendicular to the junction. Also, similar to the findings for doublets, the excitation-inhibition crosstalk of F-actin networks was not sufficient to produce co-alignment arrangement with likeliness not higher than 40% (Table G in S1 Text).

We then initialized the quadruplet in a second geometric configuration—a square (Fig 7b). In the absence of cell-cell couplings, a wider range of orientation of polarity axes was observed, presumably due to a seemingly larger degree of freedom in the configuration. As an example, we categorized the outcomes of 100 realizations of a quadruplet in square arrangement with F-actin mutual excitation-inhibition crosstalk at each cell-cell junction (last row, Table H in S1 Text). A scan of the simulations revealed that there are 5 possible configurations of polarity axes in the quadruplet: co-alignment, paired alignment, circular (clockwise or counterclockwise) alignment (S8 Movie), misalignment, or non-polarized. Overall, we found that co-alignment was rarely achieved. Of the 100 realizations, the 5 possible configuration of polarity axes were distributed as follows: 16% co-aligned, 36% paired, 6% circular, 21% misaligned, and 21% non-polarized in at least one cell (Fig 7b). However, we note that this type of coupling did not produce co-alignment in the doublets either and is only used to indicate the variety of arrangements that can emerge in more complicated domains. When instead we considered one of the 4 asymmetric Rho GTPase crosstalk interactions—up-regulation of Rac binding of Rac in one cell, and Rho in its neighbor—95% of the doublets aligned their polarity axes in a circular alignment while the remaining 5% of the doublets exhibited paired alignment. The result is sensible—the additional intercellular regions (lateral and transversal), introduce further constraints on the positioning of the front-rear axes and causing them to point either towards or away from the cross-shaped junction. This suggests that in the model, additional cell-cell or cell-environment communication are needed to ensure co-alignment rather than rotational arrangement of polarity axes in unconfined cell groups.

## Discussion

**Fig 8:**
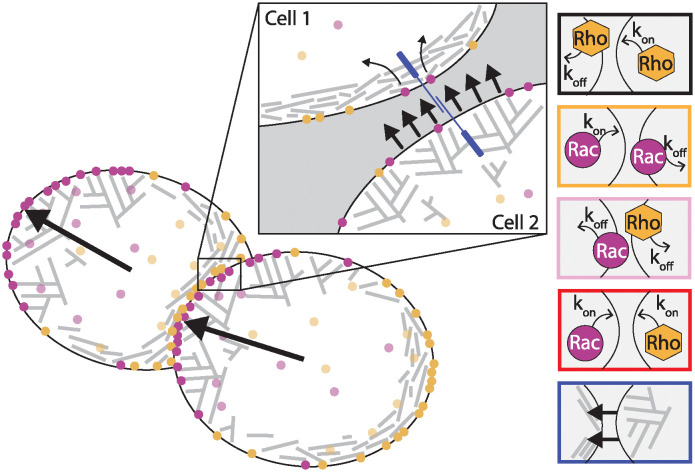
Illustration of the working principles underlying the set of intercellular interactions for collective orientation of polarity in a pair of cells. Inset illustrates our hypothesis that protrusive forces could drive enhanced dissociation of Rac in neighboring cell through mechanosensitive junction proteins.

The initiation of collective cell migration involves a complex web of signaling pathways and cytoskeletal rearrangement. In this particular cell polarization model, based on minimal assumptions, we find that only asymmetric intercellular regulation of Rho signaling or F-actin cytoskeletal dynamics can give rise to congruent orientation of polarity axes of cells in a doublet (Fig 8). We come to this conclusion by examining all possible (simple, linear) interactions at the cell-cell junction of either kinetic rates of Rho GTPases and/or F-actin network assembly. The general question of how symmetric junctional proteins, like cadherins, establish asymmetric regulation remains a rich and active area of research. Within this theoretical framework the nature of the coupling, direct or indirect, is abstracted away, and instead we think of its downstream effect on the Rho GTPase signaling pathway and/or formation of F-actin networks locally at the cell-cell junction [32, 60].

While in certain cellular systems polarity can arise from only signaling or mechanical pathways, many cells rely on the interplay between the two to robustly break symmetry to initiate locomotion. Furthermore, we are motivated to explore whether polarization is coordinated through F-actin and/or signaling dynamics in groups of cells. Therefore, we employ a mechanochemical model as the simplest polarization model where feedback between the biochemical and structural circuits gives rise to symmetry breaking without additional mechanical effects [63, 64] or unverified kinetic details [65]. The biochemical circuit has three important features: active-inactive cycling of GTPases, difference in diffusion coefficients between plasma membrane and cytosol, and conservation of mass. The model results in the emergence of multiple peaks of activity without global cellular polarization; different types of molecules segregate locally, but the clusters of the two kinds do not aggregate in the respective halves of the cell, as required for establishment of a polarity axis for migration. This suggests to us that further feedback is needed. The second circuit is for two types of dynamic F-actin networks at the cell edge, a branched protruding meshwork and an actomyosin contractile bundled network. These networks spread slowly and randomly around the cell edge, due to physical movements and tread-milling of actin filaments, and turn over while maintaining a certain equilibrium density. The nontrivial interaction between these networks is competition, such that the local density of one tends to diminish the density of another. This interaction stems both from mechanical effects and from competition for molecular resources. It was shown in [17] that this competition between two actin networks is an important part of the spontaneous polarization process, but without cell movement, the model is not able to polarize the cell, as one network will always win. Positive, bidirectional, local, and linear feedback between the structural and biochemical circuits is sufficient for spontaneous polarity [39]. Alternative feedback coupling between structural and biochemical circuits could also produce symmetry breaking in the model (Fig E in S1 Text).

Since without any cues from the environment, the orientation of the front-to-rear axis is not pre-determined, we use the model to probe the intercellular interactions that ensure front-rear axes in cell doublets point in the same direction—co-polarization. Out of the over 300 distinct pathways we scan, we find that only one type of interaction produces high probability of co-polarization of the doublet. This pathway involves asymmetric regulation across the cell-cell junction. The asymmetric regulation can be achieved through biochemical signaling—one-sided dimming/suppression of the same type of Rho GTPase across the junction, which indirectly promotes the activation (and association) of the antagonistic Rho GTPase. Essentially, the mechanism can be re-stated in the terminology of the inhibition of ‘frontness’ and ‘backness’ implicated in polarization of neutrophils and *Dictyostelium discoideum*. At the cell junction, presence of ‘frontness’ in one cell ensures diminished ‘frontness’ in the neighboring cell and, thus, ‘backness’ in the neighboring cell. Another mechanism is through enhanced activation of opposing Rho GTPases across the junction; for example, increased activation of Rac in one cell and enhanced Rho association in the neighboring cell results in co-polarization of the doublet. But the asymmetric regulation can also be achieved through F-actin dynamics—up-regulation of growth of one type of network in one cell with simultaneous up-regulation of the complementary network type in the neighboring cell. We posit that this type of interaction could arise from displacement-induced behavior not dissimilar from what has been reported in keratocytes where the forward movement of the plasma membrane engages in positive feedback with assembly of the branched F-actin network in the lamellipodium [17]. This is not an opaque finding; however, we show that, at least in this theoretical framework, only asymmetric interaction motifs, either involving biochemical signaling or F-actin dynamics, can ensure cells in the group ‘agree’ on the same axis of migration.

While the focus of our work lies in the systematic search of possible intercellular interactions motivating collective polarity at the onset of migration, our model findings are aligned with recent experimental findings. Using magnetic beads coated with cadherins, [66] demonstrated that pulling forces induce protrusions at the opposite end of the cell in both single cells and cell chains. In *Drosophila* border cells, leader-driven suppression of protrusive activity in follower cells is mediated by Rac [48]. In *Drosophila* follicular epithelial cells, Fat2 localizes to the trailing edge of each cell and promotes the formation of F-actin rich protrusions at the leading edge of the cell behind [53]. Similarly, human umbilical vein endothelial cells have been shown to have polarized Arp2/3 and VE-cadherin rich membrane protrusions, called ‘cadherin fingers’, which locally lowered actomyosin contractility in follower cells as means for tissue level organization. Our model also supports that asymmetric regulation of Rho GTPases is a universal, albeit not exclusive, pathway to negotiate front-to-rear alignment across cell groups. Additionally, we find that co-polarization can also be achieved with crosstalk between structural and biochemical circuits; either co-alignment or supracellular arrangement is a likely outcome if we assume that bundled network up-regulate Rac association in the neighboring cell and, similarly, branched network up-regulates Rho association rates (Table B in S1 Text). The assumption that branched filaments through Arp2/3 can locally up-regulate Rac binding in the cell-cell region has been experimentally observed in epithelial cells [58]. In our model, we find that these asymmetric intercellular interactions are a conserved set of co-polarization pathways even with cell-to-cell variability or with external signal bias of the kinetics of the polarity molecules in a certain direction.

In addition to the parameter constrains of the polarization model for a single cell as described in [39], we find that the asymmetric regulation of the kinetic rates or network growth rates at the intercellular region has to be strong enough to overcome the other dynamics including feedback from F-actin networks but also GTPase active-inactive cycling. It is very likely that the model performs in 2D and 3D as well as in 1D, but neither the single cell model nor the doublet model has been extended to higher dimensions. Another limitation of our model is that more complex intercellular interactions are largely not considered—these could be nonlinear in nature, involve multiple species simultaneously, or involve other mediators, such as curve-sensing proteins. Lastly, we report that our findings depend on the size of the cell-cell region relative to the cell perimeter, and, in particular, too large of a junction prevents co-orientation of the doublets (Table H in S1 Text).

The first conceptual prediction of our model is that in the absence of regulations to F-actin structures or the Rac/Rho signaling pathway by intercellular junction proteins, co-orientation of polarity axes in the same direction is lost. For cell doublets that rely on cadherins for tissue organization, if their cadherins lacked cytoplasmic domains to engage with the cytoskeleton network, the doublets would result in poor co-orientation of their polarity axes with a 25% chance of co-alignment polarization arrangement. Experiments suggest that indeed, collective cell migration is impaired or weakened upon reduced mechanical coupling between cells [67, 68]. Another conceptual biological prediction of our model is that the time for spontaneous polarization is not reduced for doublets over individual cells (Fig 6). While studies have reported on the improved persistence of polarization in groups over individual cells [69, 70], here we report on the time to break symmetry in collective groups over individuals. Our model also posits that larger cell groups require additional or more complicated crosstalk to ensure co-polarization of the group, since our 4-cell cluster in a square arrangement could give rise to paired-like or rotational-like arrangements of polarization axes (Fig 7b). In fact, experimental studies have shown collective rotational migration of a few MDCK cells on fibronectin-coated discs, and without additional guidance cues [71]. An exciting recent study, demonstrates that cells in a chain-like configuration migrate faster than cells in clusters and the authors argue that the position of the intercellular junctions play a key role in ‘negotiating’ collective polarization (and thus, migration) [72]. Lastly, and not surprisingly, our model finds it is easier to achieve supracellular arrangement over co-alignment as this is more restrictive in terms of the orientations of polarity axes in the doublet.

We do not claim that our model can predict the biological details of co-polarization of groups of any cell type. Notably, one limitation of our model comes back to the underlying single cell polarization model: It is possible for our model to rely on other forms of feedback between the biochemical and structural circuits or even solely one of the two circuits. For example, negative, instead of positive, feedback between Rac and branched actin and Rho and actomyosin, respectively, could do the job [21, 42]. We also limited the dynamics of the model to the local chemical and mechanical processes, but global mechanical effects, for example, membrane tension, could play an important role in polarization of some cell types [63]. Another paradigm for mechanochemical polarization requires transport of chemicals in the signaling framework. The key to such models is that myosin-driven flow assists the polarization of signaling proteins by mechanically triggering the formation of a stable asymmetric chemical distribution [23, 73, 74]. Our model is simpler because it does not have directional movement—either in the form of a flow, as in these models, or in the form of whole cell movement, as in [17]. More detailed and complex models have included the cell-surface adhesion dynamics or the effects of environment geometry as a mechanical component in the biochemical polarization pathway [64]. Furthermore, the model does not include many molecular players—PIP, PI3K, PTEN, cadherins, G-proteins, actin regulators—but simply conceptually captures their lumped effect on the crosstalk between Rac/Rho and actin/actomyosin. Similarly, higher order, nonlinear interactions involving Hill-type functions or interactions involving multiple polarity species are largely ignored. Instead, our model posits one of the simplest quantitative frameworks, avoiding additional assumptions, for understanding a possible mechanism for coordination of spontaneous polarization in a cell doublet prior to migration. We hope our model adds to the conversation on the effects of intercellular junction proteins on the polarity molecules and their downstream effectors.

## Supporting information

S1 MovieSimulation of a cell doublet which results in co-alignment arrangement.Heatmap plots of the branched (purple) and bundled (yellow) F-actin networks are shown inside the cell. The GTPase concentrations are plotted outside the cell membrane, with purple for Rac and yellow for Rho concentration. The Rho GTPase concentrations in cell 2 (right) are shown with transparency for visibility. The front-to-rear axis is drawn from the cell center to the median of the branched F-actin network above a threshold concentration (black arrow). The time is shown in seconds. The intercellular coupling is up-regulation of binding rates of complementary Rho GTPases, Rac in cell 1, $1000k_{\text{on}}^{\text{Rac}}\left(s_{\text{cc}}\right)$, and Rho in cell 2, $1000k_{\text{on}}^{\text{Rho}}\left(s_{\text{cc}}\right)$. The simulation parameters are as in Table A in S1 Text.(MP4)

S2 Movie Simulation of an uncoupled cell doublet with misalignment arrangement of the polarity axes. As in S1 Movie, but the doublets are uncoupled meaning that there is no interaction of either Rho GTPases or F-actin networks at the intercellular region.
(MP4)

S3 Movie Simulation of a cell doublet which results in supracellular arrangement. As in S2 Movie, but a different biochemical intercellular interaction is implemented: asymmetric up-regulation of Rac binding rates across the doublet. In cell 1, binding rate of Rac molecules is increased by 1000-fold at the cell-cell junction, but nothing is changed in cell 2.
(MP4)

S4 Movie Simulation of a cell doublet in the presence of an external stimulus. The setup is the same as in S2 Movie, namely, with the same biochemical coupling of asymmetric regulation of GTPases as the cell-cell junction. In this simulation, cell 2 is subjected to an external stimulus implemented as shown in Fig 5a. The resulting arrangement of the polarity axes in the doublet is co-alignment.
(MP4)

S5 Movie Simulation of a cell doublet’s failed response to a switch in external stimulus location. As in S4 Movie, but at time *t* = 5 seconds, the location stimulus is changed from cell 2 to cell 1 in the opposite direction. The doublet cannot re-polarize in the new direction, as cell 2 fails to establish a front through membrane localization of Rac molecules or branched F-actin network.
(MP4)

S6 Movie Simulation of doublet successfully re-polarizing in a new direction in response to a signal switch. The setup is the same as S5 Movie, but in this model realization, after the signal switch at *t* = 5, the doublet does successfully re-polarize in the new direction with co-alignment arrangement.
(MP4)

S7 Movie Simulation of spontaneous polarization in co-alignment arrangement of 4 cells started in a linear configuration. As in S2 Movie, but this time the simulation involves 4 cells, rather than 2 cells. At intercellular junctions, the interaction implemented is alternating asymmetric up-regulation of binding rates of Rho GTPases. Each cell domain moves with a constant speed in the direction of the front-to-rear axis. No additional (F-actin structure) interactions between the cells are implemented.
(MP4)

S8 Movie Simulation of spontaneous polarization resulting in clockwise rotation of 4 cells in a square configuration. The setup is the same as S7 Movie, but the cells are started in a square configuration.
(MP4)

S1 Text Model and numerical implementation details. **Figure A**: (a) Supracellular front-rear axes arrangement probabilities for enhanced binding (*k*_on_) and/or unbinding (*k*_off_) rates of Rho GTPases at the cell-cell junction. The number and box color represent the outcome probability. The numbers along the axes indicate the amplification factor, while the label indicates the rate and cell affected. Modifications in cell 1 are shown along the y-axis, and cell 2 along the x-axis. The color outline corresponds to the interaction motif in Fig 3c. White asterisks mark parameter choices where no modifications are made in one of the doublet cells yet supracellular outcome is successful. (b) Doublet simulation in supracellular arrangement. **Figure B**: Parameter sweeps for concentration dependent intercellular regulation of Rho GTPase rates. Outcome probability for co-alignment (bottom) and supracellular (top) arrangement are indicated by the number and box color. Outlined boxes highlight over 70% likeliness, and the color corresponds to a motif in Fig 3c. Dashed outlines mean not successful outcomes but previously identified as successful in Fig 3. **Figure C**: Probabilities of supracellular arrangement projected onto a 3D parameter space exploration with (a) the additive F-actin network growth rate constants as in Eq 5 and (b) concentration dependent network growth rate constants in Eq 6. The constants can take on positive, zero, or negative values. White asterisks indicate regions where mutual excitation-inhibition of the same type of F-actin structures did not produce successful outcomes for supracellular arrangement. **Figure D**: A directional bias is imposed on cell 2 due to an external stimulus which modified the dynamics of both polarity proteins Rac and Rho. Probabilities for leader-follower (supracellular) arrangement of the polarity axes of the doublet with intercellular coupling of (a) Rho GTPases (a) or (b) F-actin structures. The outlined boxes indicate over 70% likeliness for the arrangement. The color of the box outline matches the cell-cell interaction schematic in Fig 3c. **Figure E**: Single cell simulation outputs for modified couplings between Rho and bundled actomyosin network (B). (a) Top: schematic of the one-sided coupled model: only Rac proteins and the branched actin network engage in mutual local positive feedback. Bottom: we introduce additional positive feedback from Rho molecules to bundled network. (b) Schematic of the negative feedback from bundled network to Rho molecules but positive feedback from Rho to bundled network. (c) Schematic of the negative feedback from branched network (A) to Rho molecules, with positive feedback from Rho to bundled network. **Table A**: Definition and values of parameters for the mechanochemical polarity model. **Table B**: Pathways of communication between a pair of cells and the probability of a supracellular arrangement (S.), co-alignment (Co-A.), collision (C.), misalignment (Mis.), or non-polarized (N.P.) arrangement. **Table C**: Doublet polarity outcome probabilities for a subset of cell-cell coupling pathways, where cell 2 is assumed to have more responsive GTP activity over the entire domain, either through increased binding rate of Rac and/or Rho. The outcome probabilities are listed for supracellular (S.), co-alignment (Co.-A.), collision (C.), misalignment (Mis.), or non-polarized (N.P.) arrangements. **Table D**: Doublet polarity outcome probabilities for a subset of cell-cell coupling pathways, where cell 2 is assumed to have faster actin assembly dynamics over the entire domain, either through increased growth rate of branched and/or bundled network. The outcome probabilities are listed for supracellular (S.), co-alignment (Co.-A.), collision (C.), misalignment (Mis.), or non-polarized (N.P.) arrangements. **Table E**: Doublet polarity outcome probabilities for a switch in orientation of the external stimulus. For 0 ≤ *t* ≤ 5 seconds, cell 2 receives the external stimulus, but for 5 < *t* ≤ 100 seconds, cell 1 receives the stimulus in a new direction. A few cell-cell coupling pathways are tested, and the outcome likeliness is reported for doublet polarization in the new direction of the external signal (S.P.) and co-alignment arrangement (Co.-A.). **Table F**: Pathways of communication between four cells in a linear arrangement and the probability of the cells polarizing in same direction (L/R), supracellular (S.) or co-alignment (Co-A.) arrangement. **Table G**: Pathways of communication between four cells in a square arrangement and the probability of the cells polarizing in a supracellular (S.) or co-alignment (Co-A.) arrangement. Table H: Co-alignment and non-polarized (N.P.) outcomes for 100 model realizations with variations in the size of the cell-cell coupling region (as a fraction of an individual cell’s perimeter). For the intercellular coupling, 5 different biochemical or structural interaction motifs are screened.
(PDF)
