# Supplementary material for: Model supports asymmetric regulation across the intercellular junction for collective cell polarization
Source: PLoS Comput Biol. 2024 Dec 17;20(12):e1012216. doi: 10.1371/journal.pcbi.1012216 (PMC11687927; doi:10.1371/journal.pcbi.1012216)
Supplement: S1 Text — Figure A: (a) Supracellular front-rear axes arrangement probabilities for enhanced binding (kon) and/or unbinding (koff) rates of Rho GTPases at the cell-cell junction. The number and box color represent the outcome probability. The numbers along the axes indicate the amplification factor, while the label indicates the rate and cell affected. Modifications in cell 1 are shown along the y-axis, and cell 2 along the x-axis. The color outline corresponds to the interaction motif in Fig 3c. White asterisks mark parameter choices where no modifications are made in one of the doublet cells yet supracellular outcome is successful. (b) Doublet simulation in supracellular arrangement. Figure B: Parameter sweeps for concentration dependent intercellular regulation of Rho GTPase rates. Outcome probability for co-alignment (bottom) and supracellular (top) arrangement are indicated by the number and box color. Outlined boxes highlight over 70% likeliness, and the color corresponds to a motif in Fig 3c. Dashed outlines mean not successful outcomes but previously identified as successful in Fig 3. Figure C: Probabilities of supracellular arrangement projected onto a 3D parameter space exploration with (a) the additive F-actin network growth rate constants as in Eq 5 and (b) concentration dependent network growth rate constants in Eq 6. The constants can take on positive, zero, or negative values. White asterisks indicate regions where mutual excitation-inhibition of the same type of F-actin structures did not produce successful outcomes for supracellular arrangement. Figure D: A directional bias is imposed on cell 2 due to an external stimulus which modified the dynamics of both polarity proteins Rac and Rho. Probabilities for leader-follower (supracellular) arrangement of the polarity axes of the doublet with intercellular coupling of (a) Rho GTPases (a) or (b) F-actin structures. The outlined boxes indicate over 70% likeliness for the arrangement. The color of the box outline ma [file pcbi.1012216.s009.pdf]

S1 Text for: Model supports asymmetric regulation across the intercellular junction for collective cell polarization

Katherine Levandosky, Calina Copos*
* c.copos@northeastern.edu

The SI text is organized as follows: A is an overview of the mechanochemical polarization model for a single cell originally published in [1], while the extension to the cell doublet is presented in B. Numerical and computational implementation details are provided in C. Partial results of the preliminary exploratory screening for intercellular couplings for collective polarization are in D and a longer table in available online at the Zenodo repository: `https://zenodo.org/records/13716626`. Lastly, supporting results referenced in the Article are in E.

A Single cell polarization model equations

The model considers the formation of two actin networks in competition for limited molecular resources and coupled to the dynamics of membrane-bound, active Rac and Rho small Rho GTPase molecules. In the model, the polarity dynamics are on a one-dimensional circular domain representing polarity molecules on the plasma membrane and a thin volume of cytoplasm adjacent to the membrane on the circular edge of a disk-like cell spread on a flat surface. Thus, the molecular densities are localized to a circle of arc length s and circumferential length L. First, we describe the assumptions and mathematical equations of each circuit separately and then their coupling. Model parameters are provided in Table A and the implementation details below in Section C.

F-actin structural circuit. The F-actin structural model is a competition of two distinct actin networks: Arp2/3 branched (A) and actomyosin bundled (B), with the following dynamics:

1. *Autocatalytic growth*: The net growth rate of each network is proportional to local network density. This assumption is based on the processes of polymerization of existent actin filaments and of nucleation of nascent filaments by proteins binding to the existent filaments, so that the net growth becomes proportional to the existent density;

2. *Limited growth*: At high density, growth is limited due to lack of availability of molecular resources. In the case of the bundled actin network, growth could be limited due to depletion of the myosin-II motors or actin monomers, while the branched actin network growth could be limited by availability of Arp2/3 branching complexes or globular actin monomers;

3. *Competition for molecular resources*: Both networks compete for a limited cytoplasmic pool of molecular resources, such as G-actin monomers, Arp2/3 complexes, formins or myosin;

4. *Diffusive-driven redistribution of the networks along the cell boundary*: We assume, following [2], an effective diffusive spread of actin densities along the cell edge due to lateral shifts of the actin density due to filament growth and/or to physical sliding of filaments along the cell edge pulled by myosin motors.

Mathematically, based on these assumed dynamics, one arrives at the following set of non-dimensionalized PDEs [2]:

$$\frac{\partial A}{\partial t} = A - A^2 - m_0\, AB + D\Delta A, \qquad (1)$$
$$\frac{\partial B}{\partial t} = B - B^2 - m_0\, AB + D\Delta B.$$

Here, $A(s,t)$ denotes the branched F-actin network density and $B(s,t)$ represents the bundled actomyosin network density along the cell boundary parameterized by the arc length s at time t. Densities of both actin networks are defined on the 1D periodic boundary. m_0 is the non-dimensional competition parameter, and D

is the non-dimensional diffusion coefficient. Note that this effective diffusion coefficient is the result of an effective random walk of the growing ends of branched filaments along the cell edge. These filaments are growing skewed to the left and right, and thus glide along the cell edge for about a second before being capped, and then daughter filaments glide in opposite directions. For bundled filaments, the diffusion originates from the myosin-powered shuffling of the filaments along the cell edge. Respective diffusion coefficients have the same order of magnitude analyzed in [2]. Note also that we chose to model the cytoskeleton in a continuous, deterministic way, because the estimate for the number of actin on the cell edge, $N \sim 10^4$ [3] is much higher than the estimate for the number of signaling molecules on the membrane: $N \sim 10^3$ in the whole cell, of which $\sim 10\%$ is on the membrane [4].

Although Eq. 1 represent conservation laws for the two actin networks, we can also use the balance of forces to justify the mechanical nature of expressions for some parameters in this model and identify the connection to physical/mechanical forces including myosin contractile force, membrane tension, and effective friction from adhesion of the actin networks to the substrate. Specifically, small and dynamic nascent adhesions based on integrin molecules spanning the cell membrane interconnect the branched actin and the substrate. More mature focal adhesions, also integrin based but including many adaptors, force-sensing and signaling molecules, connect actomyosin bundles with themselves and the substrate. Besides the kinetic effect contributing to the term m_0AB – the competition of the branched and bundled actin networks for the same G-actin monomer pool – there are also underlying mechanical processes. In the update equation for the branched actin network (Eq. 1a), the competition term, $-m_0AB = -(m_0B)A$, describes the rate at which branched filaments are incorporated into antiparallel contractile actin bundles in the presence of myosin motors. The rate is proportional to the actomyosin density. The competition term in the update equation for bundled actomyosin network (Eq. 1b) has a similar mechanical underpinning. The expression $-m_0AB = -(m_0A)B$ is the rate of removal of the bundled actin by the flow generated by branched actin filaments growing against the membrane at the cell edge and centripetally pushing the bundles away from the edge. The rate is proportional to the branched actin density because of the force balance between the membrane tension and strength of adhesions of the bundled actin to the substrate. Assuming a viscous behavior of adhesions, the centripetal flow rate is $v = T/\zeta$ where ζ is the adhesion strength while T is the membrane tension. The membrane tension which is likely to be proportional to the density of branched filaments pushing on the membrane from within, hence the centripetal flow is proportional to the density of branched actin network [5].

Biochemical signaling circuit. We focus on the mutually exclusive interactions between Rac and Rho, two small Rho GTPases, on the plasma membrane. In the model, each GTPase molecule cycles between two states: an active GTP-bound form, bound to the plasma membrane, and an inactive GDP-bound form, freely diffusing in the cytosol. In the simulation, we only track coordinates of activated, GTP-bound, Rac and Rho molecules on the membrane along the cell edge, $x_j^{\mathrm{Rac}}(t)$ and $x_j^{\mathrm{Rho}}(t)$, respectively, where j is the index of a specific active molecule at a discrete location on circular domain at time t. Cytoplasmic concentrations of Rac and Rho are assumed to be homogeneous due to the fast diffusion in the cytoplasm. Following the rationale of the stochastic model proposed by Altschuler et al. [6], we assume five different kinds of molecular events:

1. *Spontaneous association to the membrane*: GEFs catalyze the exchange of GDP for GTP. Then, GTP-bound Rho GTPase proteins undergo a conformational change and transition to an active membrane-bound state. We model this by an association (and binding) of a respective molecule from the cytosol to a random location on the membrane at a rate of k_{on}.

2. *Spontaneous disassociation from the membrane*: GAP proteins regulate the transition of active, membrane-bound Rho GTPase into an inactive, GDP-bound, cytosolic state. This event is modeled through the removal (unbinding) of an active molecule from the membrane at a rate of k_{off}.

3. *Enhanced membrane association through activators*: Local positive feedback loops are thought to play a role in sustaining nascent Rac/Rho sites on the plasma membrane [7–10]. To model these feedback loops, we assume that a membrane-bound (active) molecule of either type (Rac or Rho) can indirectly activate and recruit a molecule of the same type to its vicinity. The rate at which one molecule recruits from the cytosol is proportional to the fraction of molecules which are still in the cytosol, with a proportionality constant of k_{fb}.

4. *Diffusion on the membrane*: Each molecule on the membrane undergoes a Brownian motion with diffusion coefficient D.

5. *Steric interaction*: In the association, recruitment, and diffusive processes, Rac and Rho proteins cannot occupy the same location in space at a given time. This assumption is based on the reported mutual antagonistic interactions between Rho GTPases [11–16].

We first outline the algorithm implementation for the Rac/Rho dynamics when the kinetic rates are constant in space. The system is initialized with 10% of the total number of signaling molecules of each type (Rac/Rho), N. These initial molecules are randomly placed along the cell membrane, ensuring that particles of different type do not spatially overlap. The number of Rac (or Rho) particles on the cell membrane, $n(t)$, evolves by a continuous-time Markov-chain process. Because the signaling dynamics will eventually be coupled to spatially-varying actin concentrations, we consider individual rather than aggregate transition rates. For each membrane-bound particle j, the time and location of the next biochemical reaction event is computed discretely. In particular, the time to the next reaction for the membrane-bound particle j is exponentially distributed with rate:

$$\lambda(n)_j = (k_{\text{off}})_j + \left(\frac{N}{n} - 1\right)\left((k_{\text{on}})_j + (k_{\text{fb}})_j \frac{n}{N}\right). \tag{2}$$

The rate $\lambda(n)_j$ should be interpreted as a reaction rate per membrane-bound particle—hence, the unbinding rate per particle is a constant k_{off}, while the binding rate is proportional to the remaining fraction of available binding spots on the cell membrane with a constant of proportionality $(k_{\text{on}}) + (k_{\text{fb}})\,\frac{n}{N}$. The spontaneous association rate per particle is constant, but the enhanced association per particle due to its implicit dynamics of enhanced recruitment is proportional to the fraction of molecules that are still in the cytosol.

After n random times based on these rates are generated, the time for the next reaction in the system is chosen as the minimum time across all active particles. Then, for each respective particle, either a disassociation event with probability $(k_{\text{off}})_j/\lambda(n)_j$, or a spontaneous association event with probability $\left(\frac{N}{n} - 1\right)(k_{\text{on}})_j/\lambda(n)_j$, or an induced association event with $\left(1 - \frac{n}{N}\right)(k_{\text{fb}})_j/\lambda(n)_j$ has occurred. If a disassociation event has occurred, the particle is removed from the membrane and added to the well-mixed homogeneous cytoplasmic pool of inactive particles. If a positive feedback–induced recruitment association event has occurred, a particle is added to the membrane from the cytoplasmic pool and its position is chosen to coincide with the position of the already membrane-bound particle. Last, for a spontaneous association event, the new particle is added from the cytoplasmic pool of inactive particles to the membrane at a randomly chosen location within the spatial segment centered at particle j. The endpoints of the spatial segment associated with particle j are at the halfway location between the nearest neighboring membrane-bound particles of the same type. Independently, this process is repeated for both Rac and Rho particles. Between Markov events, the number of membrane-bound Rac (or Rho) particles is constant, and the particles diffuse freely on the membrane. A steric repulsion is enforced between Rac and Rho polarity molecules so that the two chemicals cannot cross paths at any moment in time. The probability for the number of particles of each type can be expressed via a master equation as given in Altschuler et al. [6]. However, we note that to our knowledge, no such master equation can be easily expressed in the case of spatially-varying kinetic rates. In the presence of the mechanochemical coupling, the kinetic rates depend on position on the cell edge, and the algorithm is modified as discussed below.

Structural-chemical coupling. For the mutual coupling between F-actin structures and Rac/Rho molecules, we assume that there is a local feedback loop with a local and linear dependence on relative concentrations. The chemical rates in the signaling kinetics are no longer constant, but rather dependent on the local concentration of each respective actin network, which evolves in both space and time:

$$k^{\text{Rac}}_{\text{fb, on}}(s) = k_{\text{fb, on}}\left(1 + \beta \min[A(s), C_{\text{ss}}]\right), \tag{3}$$

$$k^{\text{Rho}}_{\text{fb, on}}(s) = k_{\text{fb, on}}\left(1 + \beta \min[B(s), C_{\text{ss}}]\right), \tag{4}$$

$$k^{\text{Rac}}_{\text{off}}(s) = k_{\text{off}}, \tag{5}$$

$$k^{\text{Rho}}_{\text{off}}(s) = k_{\text{off}}. \tag{6}$$

Here, the expressions for on and off rates are for either Rac or Rho chemical kinetics. For Rac/Rho kinetics, C denotes branched/ bundled actin network density, A or B, respectively. Thus, the induced on rate

for Rac increases with the local branched actin density, while the Rho induced on rate increases with the local bundled actin–myosin density. The off rates are constant. The strength of the coupling from actin to the polarity model is denoted by the constant of proportionality β. The minimum function in the expressions for k_{fb} and k_{on}, is a pointwise minimum function and serves to ensure that the association rates do not exceed a threshold value set by the steady state concentration C_{ss} defined in Table A.

On the reverse, the growth rate of each actin network is now an evolving parameter that depends linearly on the local amount of active or membrane-bound polarity proteins:

$$\frac{\partial A(s,t)}{\partial t} = A\left(1+\alpha\, n^{\text{Rac}}(s,t)\right) - A^2 - m_0 AB + D\Delta A \tag{7}$$

$$\frac{\partial B(s,t)}{\partial t} = B\left(1+\alpha\, n^{\text{Rho}}(s,t)\right) - B^2 - m_0 AB + D\Delta B, \tag{8}$$

where α represents the strength of the coupling from the polarity molecules to the cytoskeleton. In these expressions, $n^{\text{Rac}}(s,t)$ and $n^{\text{Rho}}(s,t)$ are the densities of Rac and Rho, respectively. Numerically, these densities are computed from the discrete locations of the respective molecules by computing at each time step a superposition of Gaussian peaks with variance and centers at their molecular locations. Their molecular locations are given by x_i^{Rac} and x_i^{Rho}.

Importantly, in the presence of the feedback from actin networks to the signaling molecules, their on/off rates depend on the spatial positions on the cell edge due to varying actin densities. Because the kinetic rates vary spatially, the individual time between events in the number of membrane-bound molecules is determined for every individual molecule according to the rates as in Eq. 2. The rates now depend on the positions of the active signaling molecules.

B Doublet extension of polarization model

The model was extended for a pair of cells by implementing two copies of the single cell model simultaneously, where each cell has its own autonomous polarity model as described in the above section. For clarity, the cell on the left is referred to as "cell 1" while the cell on the right as "cell 2." A region of cell-cell connectivity is pre-defined to be 25% of the cell membrane in both cells, (s_{cc}), where the cells can engage in intercellular interactions. The region of cell-cell contact is fixed throughout the simulation, for all simulations. The cell-cell region is centered at 270° counterclockwise from the top of the cell in cell 1 and centered at 90° in cell 2. All means of intercellular communication were implemented only in this cell-cell region by effectively changing rates in either the biochemical or structural circuit.

As an example of how intercellular communication is applied, we consider that, at the cell-cell contact region, Rac binding (association) to the plasma membrane is up-regulated in both cells. Prior to any chemical reaction or Brownian motion occurs, the binding rate $k_{\text{on}}^{\text{Rac}}$ is multiplied by an amplification factor, γ, (e.g. $\gamma = 10$) at each point within the cell-cell region, s_{cc}. Similarly, to modify the growth rate of one of the F-actin networks, the coefficient $(1+\alpha\, n^{\text{Rac}}(s,t))$ in Eq. 7 is changed to $(1+\alpha\, n^{\text{Rac}}(s,t)+\epsilon)$ where ϵ is a constant nonzero only at the discretized points in the cell-cell region.

C Numerical methods

We simulated model equations using custom codes written in MatLab (MathWorks, Natick MA). The numerical simulations carried out here were on one-dimensional periodic domains, visualized on a circle for ease. The computational domain, which represents concentrations in the plasma membrane and a thin volume of cytoplasm adjacent to the membrane, was discretized using 101 points with an averaged spatial discretization of $\Delta s = 0.1\ \mu$m. The temporal discretization was $\Delta t = 0.01$ sec and simulations were run to 30–100 seconds. Model parameters along with justifications for the choice of values are provided in supplementary material, Table A. We performed simulations using the baseline parameter values (Table A), unless otherwise indicated. The computational code is freely available online on a Github public repository: `https://github.com/CoposLab/Co-polarity` and Zenodo repository: `https://zenodo.org/records/13716626`.

Parameter	Value	Description	Reported value	Reference
L	10 μm	Length of cell	$\sim 5-20$ μm	[17]
D	0.5 μm^2/s	Effective diffusion coefficient of signaling molecules and actin	$\sim$ 0.5 μm^2/s	[6, 18, 19], [2]
m_0	2	Competition or bundling term (non-dimensionalized)		Chosen from compartment model simulations in order to give rise to quasi-stable polar solutions.
N	200	Total number of Rho GTPase molecules in cell (conserved, 100 for each type)		Chosen from Altschuler et al. [6] in order to give rise to patches.
k_{on}	0.001/s	Association rate for Rho GTPases	1.67×10^{-5}/s-0.027/s	[6, 19]
k_{fb}	1/s	Autocatalytic activation rate for Rho GTPases	0.1667/s	[6]
k_{off}	0.9/sec	Disassociation rate for Rho GTPases	1/s, 0.15/s, 0.02/s	[6, 18, 20–22]
h_{eq}	0.1	Fraction of membrane-bound Rho GTPases	2-10%	[6, 19]
ϵ	0.01 μm^2	Variance of Gaussian function used sampling Rac/Rho concentrations		
C_{crit}	1	Critical threshold actin concentration		
C_{ss}	10	Steady state actin concentration		
s_{cc}	25%	Percent of cell membrane in contact region (fixed)		
s_{per}	40%	Percent of cell membrane to receive signal		
ϵ_i	$-7.5, 0, 7.5$	Enhanced F-actin network growth rate		Chosen to be 50% of maximum observed concentration for networks.
ϵ_{ij}	$-0.9, 0, 0.9$	Enhanced F-actin network growth rate (conc. dep)		Chosen to yield highest outcome probabilities for co-alignment.

Table A: Definition and values of parameters for the mechanochemical polarity model.

The actin dynamics PDEs in Eq. 1 were solved on a 1D periodic domain using Crank-Nicolson finite difference numerical method with periodic boundary conditions. The actin networks were randomly distributed initially, with equal relative concentrations between branched and bundled networks. A modified Gillespie algorithm was used for the next reaction time for the polarity molecules. The time between Markov jumps is exponentially distributed with individual rate, as provided in Eq. 2. In between the jumps, the molecules with locations $x_i^{Rac}(t)$ and $x_i^{Rho}(t)$, where i is the index of the specific molecule, undergo Brownian motion on the membrane with diffusion coefficient d: $\Delta x = \sqrt{2d\Delta t}$. Since we enforced segregation of Rac and Rho, collisions

between a Rac molecule and a Rho molecule in the diffusive process may occur. We resolved collision events by not allowing either molecule to move into the space (interval of width Δx around a given molecule) that would result in overlap (collisions between Rac and Rac or Rho and Rho molecules are tolerated). Other more sophisticated collision resolution methods could have been employed, but for simplicity we chose this minimal dynamic. We have assessed what would happen in the absence of any such steric interaction, by running 20 simulations without any collision detection (with default values for all other parameters) and found a polarization probability of 95% (when such probability is 100% in the presence of steric interaction). In the instances of polarity establishment, the cell polarizes by actin dynamics but with less well-defined peaks in Rac and Rho concentrations. Thus, it seems that the assumed steric repulsion is helpful for the polarization (by assisting spatial segregation of Rac and Rho), but not absolutely necessary to the overall results of the model.

D Subset of screened intercellular couplings

Based on several experimental findings, we proposed a set of intercellular interaction pathways of the individual polarity circuits and evaluated their outcome in the model (Table 1). Here, we show a slightly larger set of tested hypotheses (Table B) and more are available on Zenodo repository (link above). The first three columns indicate the proposed intercellular interaction; each intercellular interaction can impact either one or both cells. The last columns of the table indicate outcome probability for all 4 arrangements: co-alignment (Co-A.), collision (C.), misalignment (Mis.), and non-polarized (N.P.) and an additional arrangement: supracellular (or leader-follower) (S.). Description of the classification of these arrangements can be found in the Methods section of the Article. Importantly, we note that the supracellular arrangement represents an overlap of the co-alignment and misalignment arrangements. The binding rate refers to k_{on}, while the unbinding rate refers to k_{off}. Nearby is a 0.1 μm distance requirement. A legend for the interaction shorthand is provided below, at the top of Table B.

**Pathway shorthand**	**Numerical implementation**
Contact inhibition of locomotion (CIL)	Elevated Rac unbinding rate and Rho binding rates (10-fold)
Co-attraction (COA)	Elevated Rac binding rate (100-fold)
Rac/Rho antagonism: Rac to Rho	Rac binding causes 10 nearby Rho molecules to unbind (same cell)
Rac/Rho antagonism: Rho to Rac	Rho binding causes 10 nearby Rac molecules to unbind (same cell)
Rac $\vdash\dashv$ Rac	Elevated Rac unbinding rate (1000-fold) proportional to nearby number Rac molecules (neighboring cell)
Rho $\vdash\dashv$ Rho	Elevated Rho unbinding rate (1000-fold) proportional to nearby number Rho (neighboring cell)
Rac $\leftrightarrow$ Rac	Elevated Rac binding rate (1000-fold) proportional to nearby number Rac (neighboring cell)
Rho $\leftrightarrow$ Rho	Elevated Rho binding rate (1000-fold) proportional to nearby number Rho (neighboring cell)
Rac $\leftrightarrow$ Rho	Elevated Rac and Rho binding rates (1000-fold) proportional to nearby number of complementary GTPase (neighboring cell)
Branched $\rightarrow$ Bundled	Elevated bundled actin growth proportional to concentration of branched actin (neighboring cell)
Bundled $\rightarrow$ Branched	Elevated branched actin growth proportional to concentration of bundled actin (neighboring cell)
Branched $\leftrightarrow$ Bundled	Elevated branched and bundled actin growth proportional to concentration of opposite species (neighboring cell) ($\epsilon_{AB} = \epsilon_{BA} > 0$)
Branched $\rightarrow$ Rho	Elevated Rho binding rate (1000-fold) proportional to branched actin concentration (neighboring cell)

**Pathway shorthand**	**Numerical implementation**
Bundled $\rightarrow$ Rac	Elevated Rac binding rate (1000-fold) proportional to bundled actin concentration (neighboring cell)
Bundled $\dashv$ Rho	Elevated Rho unbinding rate (1000-fold) proportional to bundled actin concentration (neighboring cell)
Branched $\dashv$ Rac	Elevated Rac unbinding rate (1000-fold) proportional to branched actin concentration (neighboring cell)

**Pathway**		**Outcome Probability**				
**Cell 1**	**Cell 2**	**S.**	**Co-A.**	**C.**	**Mis.**	**N.P.**
Elevated Rac binding rate (1000-fold)		0.77	0.24	0.11	0.61	0.04
Elevated Rho binding rate (1000-fold)		0.24	0.21	0	0.69	0.1
Elevated Rac unbinding rate (1000-fold)		0.17	0.18	0	0.66	0.16
Elevated Rho unbinding rate (1000-fold)		0.62	0.24	0.13	0.48	0.15
Elevated Rac binding rate (1000-fold)		0.11	0	0.62	0.29	0.09
Elevated Rho binding rate (1000-fold)		0	0	0	0.94	0.06
Elevated Rac unbinding rate (1000-fold)		0	0	0	0.69	0.31
Elevated Rho unbinding rate (1000-fold)		0.03	0	0.64	0.15	0.21
Elevated Rho binding rate (1000-fold)	Elevated Rac binding rate (1000-fold)	0.84	0.69	0	0.23	0.08
Elevated Rho unbinding rate (1000-fold)	Elevated Rac unbinding rate (1000-fold)	0.71	0.67	0	0.08	0.25
Elevated Rac binding rate (1000-fold)	Elevated Rac unbinding rate (1000-fold)	0.75	0.72	0	0.16	0.12
Elevated Rho binding rate (1000-fold)	Elevated Rho unbinding rate (1000-fold)	0.84	0.79	0	0.12	0.09
Elevated Rac and Rho binding rates (1000-fold)	Elevated Rac and Rho unbinding rates (100-fold)	0.11	0.22	0	0.35	0.43
	Elevated Rac and Rho unbinding rates (100-fold)	0.22	0.17	0	0.67	0.16
Elevated Rac binding rate (10-fold) and lowered Rho binding rate (1/1000)		0.33	0.22	0.01	0.65	0.12
Elevated Rac binding and unbinding rates (10-fold)		0	0	0	0.6	0.4
CIL		0	0.01	0	0.56	0.43
CIL and COA		0	0	0	0.67	0.33
Rac/Rho antagonism: Rac to Rho		0.43	0.25	0	0.67	0.08
Rac/Rho antagonism: Rho to Rac		0.23	0.24	0.02	0.64	0.1
Rac/Rho antagonism: Rac to Rho and Rho to Rac		0.36	0.31	0.01	0.61	0.07
Lowered Rho binding rate (1/100) proportional to Rho molecules nearby in the same cell		0.28	0.2	0.04	0.68	0.08
Lowered Rho binding rate (1/100) proportional to Rac molecules nearby in same cell		0.28	0.22	0.04	0.52	0.22
Elevated Rac and Rho unbinding rates (100-fold) proportional to same species molecules nearby in the same cell		0	0.26	0	0.28	0.46
Elevated Rho binding rate (100-fold) proportional to Rac molecules nearby in opposite cell		0.18	0.16	0	0.66	0.18
Rac $\vdash\dashv$ Rac		0	0	0	0.67	0.33
Rho $\vdash\dashv$ Rho		0.45	0.06	0.36	0.46	0.12
Rac $\vdash\dashv$ Rac and Rho $\vdash\dashv$ Rho		0.15	0.27	0	0.46	0.27

Pathway		Outcome Probability				
**Cell 1**	**Cell 2**	**S.**	**Co-A.**	**C.**	**Mis.**	**N.P.**
Rac $\leftrightarrow$ Rac		0.05	0	0.76	0.17	0.07
Rho $\leftrightarrow$ Rho		0.01	0	0	0.93	0.07
Rac $\leftrightarrow$ Rac and Rho $\leftrightarrow$ Rho		0.08	0.62	0.03	0.24	0.11
Rac $\leftrightarrow$ Rac and lowered Rho binding rate proportional to Rho molecules nearby in opposite cell		0.32	0.16	0.26	0.52	0.06
Rac $\leftrightarrow$ Rho		0.54	0.53	0	0.45	0.02
Rac $\leftrightarrow$ Rho, Rac $\vdash\dashv$ Rac and Rho $\vdash\dashv$ Rho		0.71	0.66	0	0.23	0.11
Branched $\rightarrow$ Bundled ($k_{BA} = 0.8$)		0.2	0.2	0	0.64	0.16
Bundled $\rightarrow$ Branched ($k_{AB} = 0.8$)		0.56	0.22	0.06	0.56	0.16
Branched $\leftrightarrow$ Bundled ($k_{AB} = k_{BA} = 0.8$)		0.76	0.67	0	0.2	0.13
Branched $\rightarrow$ Rho	Bundled $\rightarrow$ Rac	0.95	0.88	0	0.09	0.03
Bundled $\dashv$ Rho	Branched$\dashv$ Rac	0.72	0.66	0	0.1	0.24
Branched $\rightarrow$ Rho	Bundled $\dashv$ Rho	0.86	0.81	0	0.06	0.13
Bundled $\rightarrow$ Rac	Branched $\dashv$ Rac	0.84	0.75	0	0.11	0.14
Bundled $\rightarrow$ Rac		0.55	0.19	0.04	0.66	0.11
Bundled $\dashv$ Rho		0.52	0.05	0.12	0.57	0.26
Branched $\rightarrow$ Rho		0.31	0.21	0	0.66	0.13
Branched $\dashv$ Rac		0.03	0.01	0	0.53	0.46
Bundled $\rightarrow$ Rac and Branched $\rightarrow$ Rho		0.63	0.44	0.01	0.51	0.04
Bundled $\dashv$ Rho and Branched $\dashv$ Rac		0.03	0.17	0	0.37	0.46
Bundled $\rightarrow$ Rac, Branched $\rightarrow$ Rho, Branched $\leftrightarrow$ Bundled		0.77	0.73	0	0.16	0.11
Bundled $\rightarrow$ Rac and Branched $\leftrightarrow$ Bundled		0.83	0.65	0	0.2	0.15
Branched $\rightarrow$ Rho and Branched $\leftrightarrow$ Bundled		0.67	0.64	0	0.26	0.1
Rho $\vdash\dashv$ Rho, Rac $\vdash\dashv$ Rac, Branched $\leftrightarrow$ Bundled		0.5	0.32	0	0.54	0.14
Rac $\leftrightarrow$ Rho and Branched $\leftrightarrow$ Bundled		0.85	0.82	0	0.04	0.14
Rho $\vdash\dashv$ Rho, Rac $\vdash\dashv$ Rac, Bundled $\rightarrow$ Rac, Branched $\rightarrow$ Rho		0.97	0.8	0	0.18	0.02
Rac $\leftrightarrow$ Rho, Bundled $\rightarrow$ Rac, Branched $\rightarrow$ Rho		0.03	0.02	0	0.97	0.01
Rho $\vdash\dashv$ Rho, Rac $\vdash\dashv$ Rac, Rac $\leftrightarrow$ Rho, Bundled $\rightarrow$ Rac, Branched $\rightarrow$ Rho		0.92	0.85	0	0.13	0.02
Rho $\vdash\dashv$ Rho, Rac $\vdash\dashv$ Rac, Rac $\leftrightarrow$ Rho, Branched $\leftrightarrow$ Bundled		0.73	0.7	0	0.21	0.09
Rho $\vdash\dashv$ Rho, Rac $\vdash\dashv$ Rac, Rac $\leftrightarrow$ Rho, Bundled $\rightarrow$ Rac, Branched $\rightarrow$ Rho, Branched $\leftrightarrow$ Bundled		0.97	0.92	0	0.07	0.01

Table B: Pathways of communication between a pair of cells and the probability of a supracellular arrangement (S.), co-alignment (Co-A.), collision (C.), misalignment (Mis.), or non-polarized (N.P.) arrangement.

For interactions involving small Rho GTPases, the information in the table indicates which polarity molecule is impacted (Rac or Rho), which kinetic rate is adjusted (k_{on} or k_{off}), and the amplification factor. For the interaction involving branched F-actin networks at the cell-cell junction, the model equations are as in Eqs. (3)-(4) with rates as in Eq. (5), namely $\epsilon_A^i = 7.5, \epsilon_B^i = 0$ for $i = 1, 2$. For the interaction involving the mechanochemical crosstalk, the binding rates of GTPases are multiplied by a constant as well as the corresponding actin network concentration; for example, the Rac binding rate in cell 1 becomes $\left(k_{\text{on}}^{\text{Rac}}\right)_1 = 100 \left(k_{\text{on}}^{\text{Rac}}\right)_1 B_2$, where B_2 denotes the concentration of bundled network in cell 2. For the interaction involving mutual Rac/Rho antagonism, a few different implementations were tested: binding of a Rac molecule causes nearby Rho molecules to unbind (with variable number of Rho molecules, 1 or 10, binding of a Rho molecule causes nearby Rac molecules to unbind (either 1 or 10 Rac molecules), or both all within a neighborhood of 0.1μm. Of these interactions, we chose the interaction which gave the most favorable results with respect to likeliness for co-alignment or supracellular arrangement. In this interaction, in the cell-cell region, within an individual cell, the random association of a Rac molecule to a particular randomly chosen position on the plasma membrane, causes the dissociation of at most one Rho molecule, if it falls within a neighborhood of 0.1 μm. Lastly, contact inhibition of locomotion (CIL) and/or coattraction

(COA) are implemented as described in [23]. Namely, at the cell-cell junction, CIL is interpreted to impact Rho GTPases by increasing the baseline value for the unbinding rate of Rac and simultaneously, the baseline value for the binding rate of Rho in both cells ($10k_{\mathrm{off}}^{\mathrm{Rac}}$ and $10k_{\mathrm{on}}^{\mathrm{Rho}}$), while COA is enacted by increasing the binding rate of Rac only ($10k_{\mathrm{on}}^{\mathrm{Rac}}$).

E Additional results referenced in the text

Motifs that support supracellular (leader-follower) outcome with biochemical intercellular crosstalk: As reported for the co-alignment arrangement (Fig 3), the same 4 types of asymmetric Rac/Rho regulation also give rise to high likeliness for supracellular arrangement (Fig A). The motifs are the same as those identified in the co-alignment case, but for a wider range of amplification factors. As an example, for supracellular arrangement, it is sufficient for Rac binding to be locally up-regulated in one cell only, while the conditions for the neighboring cell – either up-regulated Rho binding or Rac unbinding – can be relaxed (white asterisks, Fig Aa).

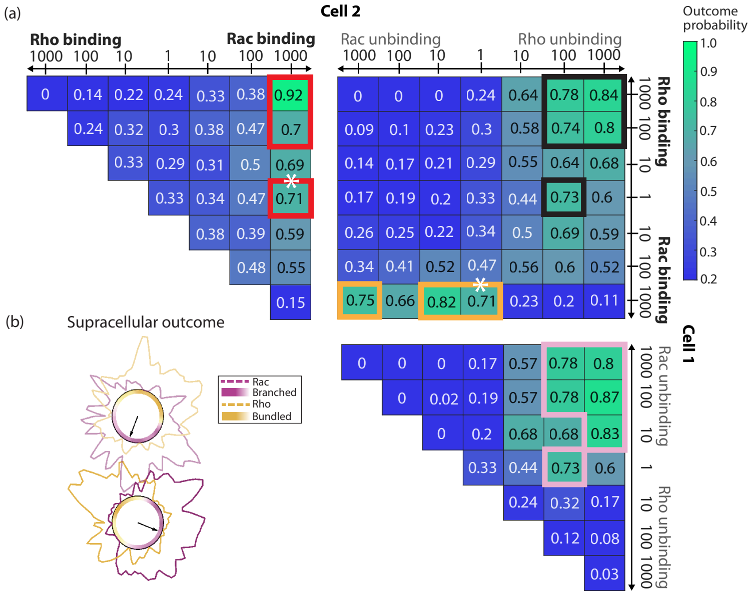

Figure A: (a) Supracellular front-rear axes arrangement probabilities for enhanced binding (k_{on}) and/or unbinding (k_{off}) rates of Rho GTPases at the cell-cell junction. The number and box color represent the outcome probability. The numbers along the axes indicate the amplification factor, while the label indicates the rate and cell affected. Modifications in cell 1 are shown along the y-axis, and cell 2 along the x-axis. The color outline corresponds to the interaction motif in Fig 3c. White asterisks mark parameter choices where no modifications are made in one of the doublet cells yet supracellular outcome is successful. (b) Doublet simulation in supracellular arrangement.

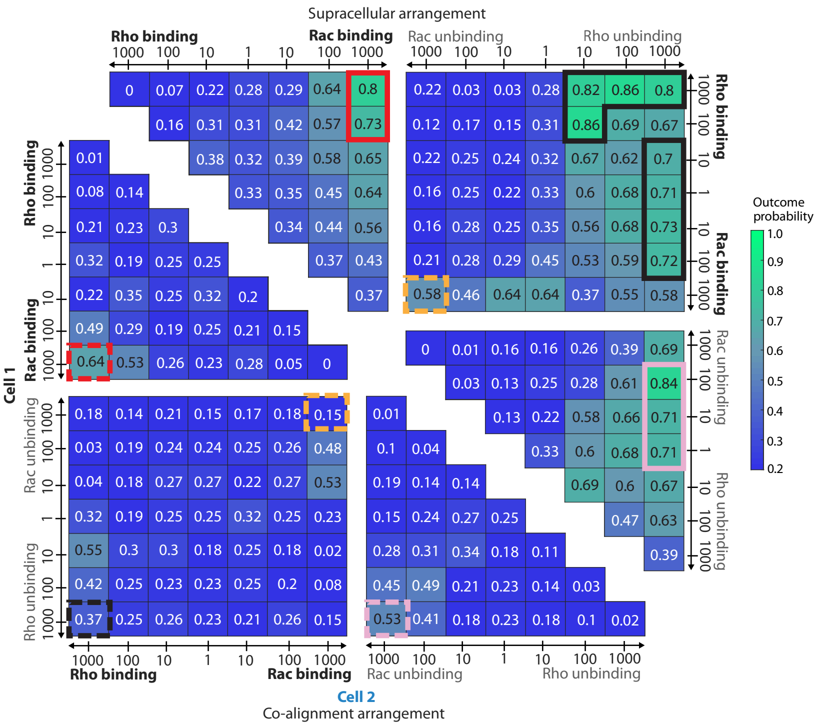

Figure B: Parameter sweeps for concentration dependent intercellular regulation of Rho GTPase rates. Outcome probability for co-alignment (bottom) and supracellular (top) arrangement are indicated by the number and box color. Outlined boxes highlight over 70% likeliness, and the color corresponds to a motif in Fig 3c. Dashed outlines mean not successful outcomes but previously identified as successful in Fig 3.

Results for concentration-dependent modulation of Rac/Rho kinetic rates: In the Article, we report that if Rac/Rho binding rates are enhanced at the intercellular region in a concentration-dependent manner, the co-alignment outcome cannot be ensured with a larger probability than 64% (Fig B). Here, we show the simulation outputs for both co-alignment (bottom, Fig B) and supracellular arrangement (top, Fig B). The concentration dependent interaction was implemented by multiplying the appropriate kinetic rate by an amplification factor and total amount of molecules in the neighboring cell engaged in that specific interaction pathway. For example, if the original intercellular interaction was enhanced binding of complementary Rho GTPases with $1000k_{\text{on}}^{\text{Rac}}$ in cell 1 and $1000k_{\text{off}}^{\text{Rho}}$ in cell 2, the concentration dependent version would be $1000k_{\text{on}}^{\text{Rac}}\, n_{\text{Rho}}^{\text{cell 2}}$ in cell 1 and $1000k_{\text{off}}^{\text{Rho}}\, n_{\text{Rac}}^{\text{cell 1}}$ in cell 2. Here, $n_{\text{Rac}}^{\text{cell 1}}$ is the total number of active, membrane-bound Rac molecules, while $n_{\text{Rho}}^{\text{cell 2}}$ is the total number of active, membrane-bound Rho molecules in a small neighborhood around the neighboring point along the cell membrane in cell 1 and 2, respectively.

Notably, in the supracellular outcome, only three of the 4 motifs that were found successful for concentration independent enhancement of kinetic rates, do produce high likeliness of this outcome; neither one of them produces successful outcomes for co-alignment arrangement.

Motifs that support supracellular (leader-follower) outcome with F-actin structural intercellular crosstalk: Compared to Fig 4, the parameter range that gives rise to supracellular arrangement of front-rear axes is larger than for co-alignment arrangement (Fig C); meaning that it is easier to ensure leader-follower than co-polarization arrangement through interactions of F-actin structures. Yet again, concentration independent changes in growth rates (Fig Ca) are preferred over concentration dependent ones (Fig Cb). The motif underpinning these successful interactions is reciprocal excitation of complementary F-actin structures. For example, in Fig Cb, the successful interactions all involve $\epsilon_{AB} > 0$ meaning that the presence of branched network in one cell enhances the growth of bundled network in the other cell.

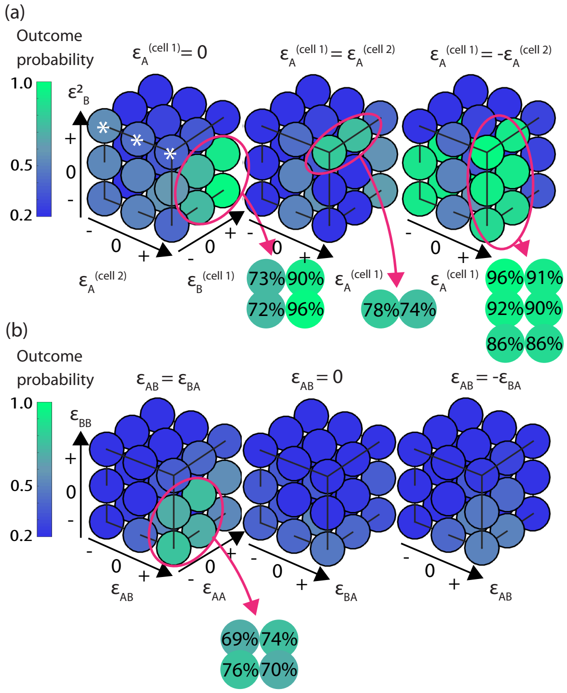

Figure C: Probabilities of supracellular arrangement projected onto a 3D parameter space exploration with (a) the additive F-actin network growth rate constants as in Eq. 5 and (b) concentration dependent network growth rate constants in Eq. 6. The constants can take on positive, zero, or negative values. White asterisks indicate regions where mutual excitation-inhibition of the same type of F-actin structures did not produce successful outcomes for supracellular arrangement.

Probability outcomes for cell-to-cell variability in the polarity model parameters: The simulation outcomes with cell-to-cell variability in the polarity dynamics are in Table C (variability in the biochemical circuit rates) and Table D (variability in the F-actin structural circuit rates). The type of cell-to-cell variability introduced in cell 2 is specified in the first column of Tables C-D. The second and third columns indicate the intercellular interaction pathway. The resulting outcome probabilities (out of 100 realizations for each interaction) are listed in the last columns of the Tables.

In the case of more responsive GTPase activity, we find that the favorable interactions that produce either supracellular or co-alignment arrangement with over 70% likeliness are the same as without cell-to-cell variability, namely asymmetric crosstalk of Rho GTPases or crosstalk of F-actin networks (Table C). Next, we consider the case that cell-to-cell variability is due to F-actin dynamics, namely higher baseline growth rates for either branched or bundled network or both; the change in the growth rate is implemented using Eqs. 5 with values for ϵ_A, ϵ_B as in Table D. The cell-cell couplings that produce high likeliness of supracellular or co-alignment arrangement are qualitatively the same as without cellular variability, although co-orientation (co-alignment) is achieved with much lower probability.

	**Pathway**		**Outcome Probability**				
**Cell 2**	**Cell 1 (cell-cell region)**	**Cell 2 (cell-cell region)**	**S.**	**Co-A.**	**C.**	**Mis.**	**N.P.**
$10k_{\text{on}}^{\text{Rac}}$	Uncoupled		0.3	0.2	0.02	0.66	0.12
	Elevated Rac binding rate (1000-fold)	Elevated Rac unbinding rate (1000-fold)	0.87	0.77	0	0.11	0.12
	Elevated Rho binding rate (1000-fold)	Elevated Rac binding rate (1000-fold)	0.91	0.85	0	0.08	0.07

	Pathway		Outcome Probability				
Cell 2	Cell 1 (cell-cell region)	Cell 2 (cell-cell region)	S.	Co-A.	C.	Mis.	N.P.
	Elevated Rho binding rate (1000-fold)	Elevated Rho unbinding rate (100-fold)	0.83	0.76	0	0.08	0.16
	Elevated Rho unbinding rate (1000-fold)	Elevated Rac unbinding rate (1000-fold)	0.9	0.8	0	0.14	0.06
	Elevated Rho unbinding rate (1000-fold)		0.67	0.26	0.15	0.49	0.1
	Elevated Rho unbinding rate (100-fold)		0.08	0	0.67	0.17	0.16
	Elevated Rac binding rate (1000-fold)	Elevated Rac binding rate (10-fold)	0.6	0.1	0.26	0.58	0.06
	CIL and COA		0.18	0.22	0	0.59	0.19
	Rac/Rho antagonism: Rac to Rho		0.43	0.29	0.01	0.67	0.03
	Branched ↔ Bundled ($\epsilon_{AB} = \epsilon_{BA} > 0$)		0.74	0.69	0	0.16	0.15
$10k_{\text{on}}^{\text{Rho}}$	Uncoupled		0.37	0.18	0.03	0.63	0.16
	Elevated Rac binding rate (1000-fold)	Elevated Rac unbinding rate (1000-fold)	0.68	0.64	0	0.08	0.28
	Elevated Rho binding rate (1000-fold)	Elevated Rac binding rate (1000-fold)	0.85	0.78	0	0.16	0.06
	Elevated Rho binding rate (1000-fold)	Elevated Rho unbinding rate (100-fold)	0.79	0.72	0.01	0.11	0.16
	Elevated Rho unbinding rate (1000-fold)	Elevated Rac unbinding rate (1000-fold)	0.83	0.78	0	0.07	0.15
	Elevated Rho unbinding rate (1000-fold)		0.57	0.15	0.15	0.56	0.14
	Elevated Rho unbinding rate (100-fold)		0.07	0	0.62	0.16	0.22
	Elevated Rac binding rate (1000-fold)	Elevated Rac binding rate (10-fold)	0.71	0.24	0.13	0.6	0.03
	CIL and COA		0	0.02	0	0.59	0.39
	Rac/Rho antagonism: Rac to Rho		0.4	0.26	0.01	0.59	0.14
	Branched ↔ Bundled ($\epsilon_{AB} = \epsilon_{BA} > 0$)		0.74	0.52	0	0.3	0.18
$10k_{\text{on}}^{\text{Rac}}$ and $10k_{\text{on}}^{\text{Rho}}$	Uncoupled		0.34	0.21	0.03	0.68	0.08
	Elevated Rac binding rate (1000-fold)	Elevated Rac unbinding rate (1000-fold)	0.7	0.65	0	0.13	0.22
	Elevated Rho binding rate (1000-fold)	Elevated Rac binding rate (1000-fold)	0.96	0.85	0	0.11	0.04
	Elevated Rho binding rate (1000-fold)	Elevated Rho unbinding rate (100-fold)	0.84	0.75	0	0.11	0.14
	Elevated Rho unbinding rate (1000-fold)	Elevated Rac unbinding rate (1000-fold)	0.82	0.73	0	0.14	0.13
	Elevated Rho unbinding rate (1000-fold)		0.59	0.2	0.15	0.46	0.19
	Elevated Rho unbinding rate (100-fold)		0.06	0	0.61	0.19	0.2
	Elevated Rac binding rate (1000-fold)	Elevated Rac binding rate (10-fold)	0.63	0.13	0.22	0.6	0.05
	CIL and COA		0.2	0.2	0	0.53	0.27
	Rac/Rho antagonism: Rac to Rho		0.39	0.24	0.02	0.68	0.06
	Branched ↔ Bundled ($\epsilon_{AB} = \epsilon_{BA} > 0$)		0.73	0.58	0	0.27	0.15

Table C: Doublet polarity outcome probabilities for a subset of cell-cell coupling pathways, where cell 2 is assumed to have more responsive GTP activity over the entire domain, either through increased binding rate of Rac and/or Rho. The outcome probabilities are listed for supracellular (S.), co-alignment (Co.-A.), collision (C.), misalignment (Mis.), or non-polarized (N.P.) arrangements.

	Pathway		Outcome Probability				
**Cell 2**	**Cell 1 (cell-cell region)**	**Cell 2 (cell-cell region)**	**S.**	**Co-A.**	**C.**	**Mis.**	**N.P.**
$\epsilon_A = 1$	Uncoupled		0.3	0.22	0.02	0.69	0.07
	Elevated Rac binding rate (1000-fold)	Elevated Rac unbinding rate (1000-fold)	0.74	0.71	0	0.25	0.04
	Elevated Rho binding rate (1000-fold)	Elevated Rac binding rate (1000-fold)	0.77	0.61	0	0.38	0.01
	Elevated Rho binding rate (1000-fold)	Elevated Rho unbinding rate (100-fold)	0.59	0.47	0	0.14	0.39
	Elevated Rho unbinding rate (1000-fold)	Elevated Rac unbinding rate (1000-fold)	0.93	0.88	0	0.07	0.05
	Elevated Rho unbinding rate (1000-fold)		0.71	0.24	0.14	0.54	0.08
	Elevated Rho unbinding rate (100-fold)		0.04	0	0.44	0.11	0.45
	Elevated Rac binding rate (1000-fold)	Elevated Rac binding rate (10-fold)	0.64	0.18	0.12	0.65	0.05
	CIL and COA		0.13	0.2	0	0.79	0.01
	Rac/Rho antagonism: Rac to Rho		0.46	0.21	0.04	0.73	0.02
	Branched $\leftrightarrow$ Bundled ($\epsilon_{AB} = \epsilon_{BA} > 0$)		0.68	0.53	0	0.37	0.1
$\epsilon_B = 1$	Uncoupled		0.26	0.2	0.03	0.63	0.14
	Elevated Rac binding rate (1000-fold)	Elevated Rac unbinding rate (1000-fold)	0.48	0.39	0	0.18	0.43
	Elevated Rho binding rate (1000-fold)	Elevated Rac binding rate (1000-fold)	0.67	0.52	0	0.46	0.02
	Elevated Rho binding rate (1000-fold)	Elevated Rho unbinding rate (100-fold)	0.97	0.77	0	0.23	0
	Elevated Rho unbinding rate (1000-fold)	Elevated Rac unbinding rate (1000-fold)	0.57	0.51	0	0.07	0.42
	Elevated Rho unbinding rate (1000-fold)		0.61	0.18	0.14	0.47	0.21
	Elevated Rho unbinding rate (100-fold)		0.11	0	0.65	0.25	0.1
	Elevated Rac binding rate (1000-fold)	Elevated Rac binding rate (10-fold)	0.63	0.27	0.13	0.5	0.1
	CIL and COA		0.04	0.15	0	0.61	0.24
	Rac/Rho antagonism: Rac to Rho		0.49	0.29	0.07	0.54	0.1
	Branched $\leftrightarrow$ Bundled ($\epsilon_{AB} = \epsilon_{BA} > 0$)		0.67	0.53	0	0.24	0.23
$\epsilon_A = 1$ and $\epsilon_B = 1$	Uncoupled		0.39	0.18	0.04	0.78	0
	Elevated Rac binding rate (1000-fold)	Elevated Rac unbinding rate (1000-fold)	0.71	0.68	0	0.19	0.13
	Elevated Rho binding rate (1000-fold)	Elevated Rac binding rate (1000-fold)	0.79	0.59	0	0.41	0
	Elevated Rho binding rate (1000-fold)	Elevated Rho unbinding rate (100-fold)	0.93	0.73	0	0.25	0.02
	Elevated Rho unbinding rate (1000-fold)	Elevated Rac unbinding rate (1000-fold)	0.9	0.82	0	0.09	0.09
	Elevated Rho unbinding rate (1000-fold)		0.69	0.21	0.2	0.56	0.03
	Elevated Rho unbinding rate (100-fold)		0.08	0	0.67	0.23	0.1
	Elevated Rac binding rate (1000-fold)	Elevated Rac binding rate (10-fold)	0.57	0.2	0.13	0.66	0.01
	CIL and COA		0.15	0.15	0	0.84	0.01
	Rac/Rho antagonism: Rac to Rho		0.44	0.27	0.01	0.71	0.01
	Branched $\leftrightarrow$ Bundled ($\epsilon_{AB} = \epsilon_{BA} > 0$)		0.79	0.67	0	0.17	0.16

	Pathway		Outcome Probability				
Cell 2	Cell 1 (cell-cell region)	Cell 2 (cell-cell region)	S.	Co-A.	C.	Mis.	N.P.

Table D: Doublet polarity outcome probabilities for a subset of cell-cell coupling pathways, where cell 2 is assumed to have faster actin assembly dynamics over the entire domain, either through increased growth rate of branched and/or bundled network. The outcome probabilities are listed for supracellular (S.), co-alignment (Co.-A.), collision (C.), misalignment (Mis.), or non-polarized (N.P.) arrangements.

Model outputs for supracellular arrangement in the doublet with an applied external stimulus: Fig D is the extension of Fig 5 in the Article for supracellular, rather than co-alignment, arrangement of polarity axes. Successful outcomes are produced by all four motifs of asymmetric regulation of Rho GTPases (Fig Da), and mutual excitation of complementary F-actin structures at the intercellular junction (Fig Db).

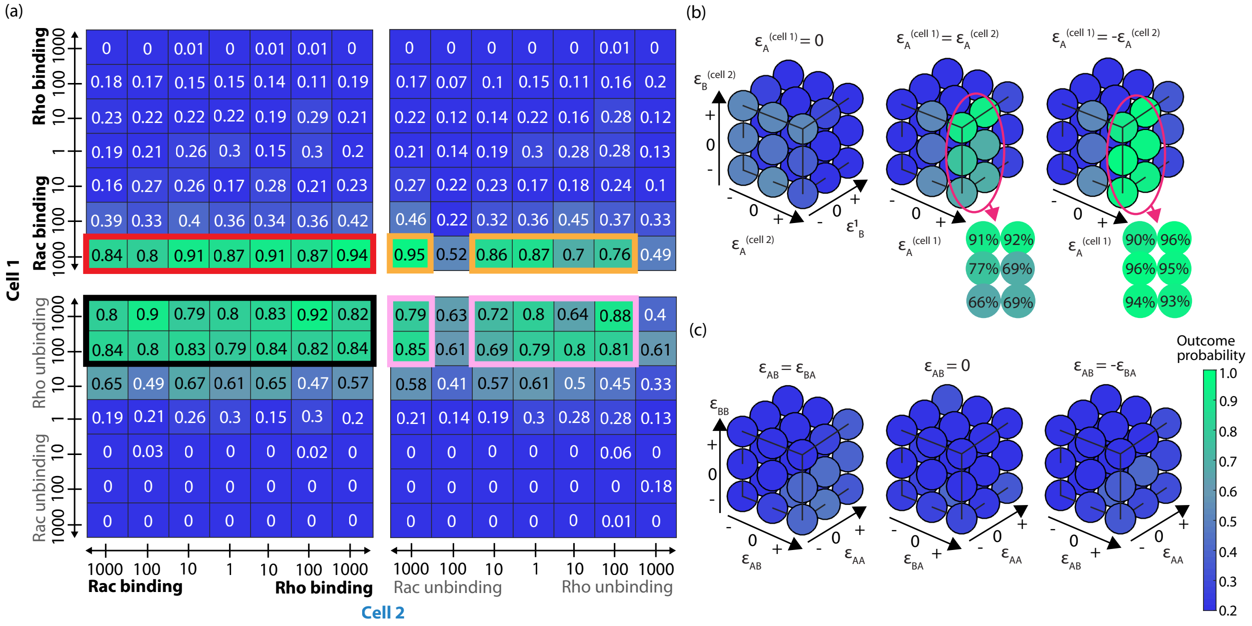

Figure D: A directional bias is imposed on cell 2 due to an external stimulus which modified the dynamics of both polarity proteins Rac and Rho. Probabilities for leader-follower (supracellular) arrangement of the polarity axes of the doublet with intercellular coupling of (a) Rho GTPases (a) or (b) F-actin structures. The outlined boxes indicate over 70% likeliness for the arrangement. The color of the box outline matches the cell-cell interaction schematic in Fig 3c.

Results for changes in the direction of the external stimulus: Table E contains the results for re-polarization of the doublet in a new direction due to a change in the external stimulus direction by 180 degrees where cell 1, rather than cell 2, is now subject to the external stimulus. For $0 \leq t \leq 5$ seconds, cell 2 receives a signal at 225 degrees from the vertical. Then, for $5 < t \leq 40$ seconds, the old signal is removed, and a new signal direction is introduced to cell 1 at 45 degrees from the vertical. The polarization outcomes are reported at the end of the simulation, $t = 40$ seconds. We find that none of the tested cell-cell interactions result in successful outcomes for co-alignment, but a couple do polarize in the direction of the new signal orientation with high fidelity for both cells – termed signal-polarized (S.P.). The difference between co-alignment and both cells pointing towards the signal just comes from the fact that the signal is 'wider' than the angle we require for co-alignment.

Model outputs for polarization of 4-cell clusters: Finally, the polarization outcomes for groups of 4 cells initiated in two different geometric arrangements (linear/chain and square) are recapitulated in Tables F and G. The first four columns indicate the intercellular coupling implemented at all cell-cell junctions, and

the last two columns report same direction (left or right) and co-alignment outcome probabilities. Notably, the same direction is a less constrained condition on the dot product between the polarity vectors, hence the higher probability outcomes when compared to co-alignment arrangement. The pathways included in the subset of intercellular interactions include: uncoupled, the 4 asymmetric crosstalk of Rho GTPases, and the asymmetric crosstalk of F-actin networks. The results are discussed in the Article.

**Pathway**		**Outcome Probability**	
**Ext. signal nonexposed cell**	**Ext. signal exposed cell**	**S.P.**	**Co-A.**
Uncoupled		0.03	0.03
Elevated Rac binding rate (1000-fold)	Elevated Rho binding rate (1000-fold)	0.16	0.12
Elevated Rac binding rate (1000-fold)	Elevated Rac unbinding rate (1000-fold)	0.14	0.11
Conc-dep. elevated Rho unbinding rate (1000-fold)	Conc-dep. elevated Rac unbinding rate (10-fold)	0.79	0.55
Up-regulated branched $\left(\epsilon_A^{(\text{cell 1})} > 0\right)$	Up-regulated bundled $\left(\epsilon_B^{(\text{cell 2})} > 0\right)$	0.38	0.18
Elevated Rho unbinding rate (1000-fold)	Elevated Rho binding rate (1000-fold)	0.75	0.45
Up-regulated branched $\left(\epsilon_A^{(\text{cell 1})} > 0\right)$ Down-regulated bundled $\left(\epsilon_B^{(\text{cell 1})} < 0\right)$	Up-regulated bundled $\left(\epsilon_B^{(\text{cell 2})} > 0\right)$ Down-regulated branched $\left(\epsilon_A^{(\text{cell 2})} < 0\right)$	0.3	0.16

Table E: Doublet polarity outcome probabilities for a switch in orientation of the external stimulus. For $0 \leq t \leq 5$ seconds, cell 2 receives the external stimulus, but for $5 < t \leq 100$ seconds, cell 1 receives the stimulus in a new direction. A few cell-cell coupling pathways are tested, and the outcome likeliness is reported for doublet polarization in the new direction of the external signal (S.P.) and co-alignment arrangement (Co.-A.).

**Pathway**				**Outcome Probability**		
**Cell 1**	**Cell 2**	**Cell 3**	**Cell 4**	**L/R**	**S.**	**Co-A.**
Uncoupled				0.13	0.04	0.01
Elevated Rho binding rate (1000-fold) (left contact regions) Elevated Rac binding rate (1000-fold) (right contact regions)				0.96	0.89	0.68
Elevated Rho unbinding rate (1000-fold) (left contact regions) Elevated Rac unbinding rate (1000-fold) (right contact regions)				0.85	0.81	0.64
Elevated Rac unbinding rate (1000-fold) (left contact regions) Elevated Rac binding rate (1000-fold) (right contact regions)				0.87	0.82	0.65
Elevated Rho binding rate (1000-fold) (left contact regions) Elevated Rho unbinding rate (1000-fold) (right contact regions)				0.87	0.8	0.56
Branched $\leftrightarrow$ Bundled ($\epsilon_{AB} = \epsilon_{BA} > 0$, $\epsilon_{AA} = \epsilon_{BB} = 0$)				0.37	0.36	0.16

Table F: Pathways of communication between four cells in a linear arrangement and the probability of the cells polarizing in same direction (L/R), supracellular (S.) or co-alignment (Co-A.) arrangement.

Results for changes in the size of the intercellular junction: In Table H, we report the probabilities of co-alignment and non-polarized arrangement with variations in cell-cell junction region from 12.5% to 25% (default) to 50% of a cell's perimeter for 5 couplings including asymmetric regulation of Rho GTPases binding or unbinding rates (Fig 3c) and assembly of complementary cytoskeletal networks ($\epsilon_A^2 = \epsilon_B^1 > 0$, $\epsilon_A^1 = 0$, $\epsilon_B^2 < 0$ case in Fig 4b). For the first four schemes involving Rho GTPase regulation, we find that the co-alignment probability is maximal when 25% of the cell perimeter is in contact with its neighboring cell. This is sensible since we expect a biphasic response – too little interaction should recapitulate the uncoupled case, while too much interaction could result in non-polarized cells. For up-regulation of Rac unbinding rates, we see loss of polarized solutions. The doublet co-orientation is improved with regulation of the assembly of the cytoskeletal networks over a larger spatial region. Within our model framework, this makes sense, as larger spatial support for growth rates improves the quasi-stability of the co-existence solution of the modified Lotka-Volterra equations [24].

An alternative structural-biochemical feedback in the single cell model Lastly, we report that in our system, in the absence of even one direction of the dual coupling, the stability of the polarized solution is lost (Fig Ea). We ran simulations with only Rac informing the actin module. This was achieved by completely decoupling the Rho dynamics from bundled actomyosin network; by setting $\alpha, \beta = 0$ for Rho and bundled actomyosin

Pathway				Outcome Probability	
**Cell 1**	**Cell 2**	**Cell 3**	**Cell 4**	**S.**	**Co-A.**
Uncoupled				0.15	0.02
Elevated Rho binding rate (1000-fold) (alternate contact regions)<br>Elevated Rac binding rate (1000-fold) (alternate contact regions)				0.15	0
Elevated Rho binding rate (1000-fold) (alternate contact regions)<br>Elevated Rho unbinding rate (1000-fold) (alternate contact regions)				0.46	0
Branched ↔ Bundled ($\epsilon_{AB} = \epsilon_{BA} > 0$, $\epsilon_{AA} = \epsilon_{BB} = 0$)				0.02	0.13

Table G: Pathways of communication between four cells in a square arrangement and the probability of the cells polarizing in a supracellular (S.) or co-alignment (Co-A.) arrangement.

**Intercellular coupling**	**12.5%**		**25%**		**50%**	
	Co-align	N.P.	Co-align	N.P.	Co-align	N.P.
Rac/Rho up-regulation	0.63	0	0.87	0.06	0.5	0.01
Rac/Rho down-regulation	0.57	0.08	0.74	0.2	0.06	0.94
Asymmetric Rac regulation	0.62	0.04	0.72	0.12	0.03	0.94
Asymmetric Rho regulation	0.71	0.1	0.79	0.09	0.58	0
Cytoskeletal regulation	0.66	0	0.81	0.01	0.95	0.05

Table H: Co-alignment and non-polarized (N.P.) outcomes for 100 model realizations with variations in the size of the cell-cell coupling region (as a fraction of an individual cell's perimeter). For the intercellular coupling, 5 different biochemical or structural interaction motifs are screened.

concentration. The outcome was a non-polarized cell in 50 out of 50 realizations of the model. Next, we kept three out of the four coupling terms and turned off the positive feedback from bundled actin network to Rho molecules by setting $\beta = 0$ for the signaling rates pertaining to Rho molecules. Again, the outcome was a non-polarized cell in 50 out of 50 realizations of the model.

Next, we implemented negative feedback between Rho and actin in two ways while maintaining the bidirectional positive feedback between Rac and branched actin network as described in the paper. In one scenario (Fig Eb), we assume that bundled network induced negative feedback on Rho, which was achieved with fixed binding reaction rates ($k_{\text{on, fb}}$), but spatially dependent unbinding rate of the form: $k_{\text{off}}^{\text{Rho}} = (1 + \beta \min[B(s), C_{ss}])$ where C_{ss} is the steady state concentration. The minimum function is a pointwise minimum function and serves to ensure that the disassociation rate does not exceed a threshold value set by the steady state concentration C_{ss} (value and justification provided in Table S1). In this case, the outcome was non-polarized cell in 50 out of 50 independent runs of the model. In the second scenario, we implemented that branched network (A), instead of bundled network, induces negative feedback on Rho locally with $k_{\text{off}}^{\text{Rho}} = (1 + \beta \min[A(s), C_{ss}])$ (Fig Ec). The outcome was 22 polarized cells of 50 runs of the model ($\alpha, \beta = 2$), but with change of parameters produced 46 polarized cells out of 50 runs ($\alpha = 3.6, \beta = 0.4$ in the A-to-Rho pathway only). This finding suggests to us that our model does support negative feedback as long as it is between branched (protrusive) F-actin network and Rho to replace the positive feedback from bundled actomyosin to Rho.

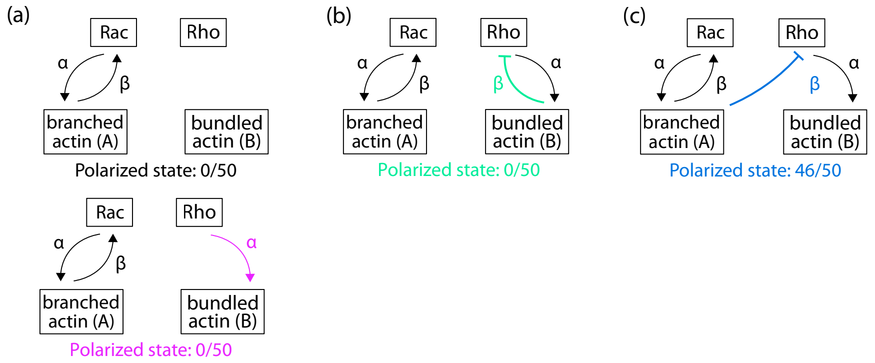

Figure E: Single cell simulation outputs for modified couplings between Rho and bundled actomyosin network (B). (a) Top: schematic of the one-sided coupled model: only Rac proteins and the branched actin network engage in mutual local positive feedback. Bottom: we introduce additional positive feedback from Rho molecules to bundled network. (b) Schematic of the negative feedback from bundled network to Rho molecules but positive feedback from Rho to bundled network. (c) Schematic of the negative feedback from branched network (A) to Rho molecules, with positive feedback from Rho to bundled network.

Bibliography

[1] Copos C, Mogilner A. A hybrid stochastic–deterministic mechanochemical model of cell polarization. Mol Biol Cell. 2020;31:1637–1649. doi:10.1091/mbc.E19-09-0549.

[2] Lomakin AJ, Lee KC, Han SJ, Bui DA, Davidson M, Mogilner A, Danuser G. Competition for actin between two distinct F-actin networks defines a bistable switch for cell polarization. Nat Cell Biol. 2015 Nov;17(11):1435-45. doi: 10.1038/ncb3246.

[3] Abraham VC, Krishnamurthi V, Taylor DL, Lanni F. The actin-based nanomachine at the leading edge of migrating cells. Biophys J. 1999 Sep;77(3):1721-32. doi: 10.1016/S0006-3495(99)77018-9.

[4] Walther GR, Marée AF, Edelstein-Keshet L, Grieneisen VA. Deterministic versus stochastic cell polarisation through wave-pinning. Bull Math Biol. 2012 Nov;74(11):2570-99. doi: 10.1007/s11538-012-9766-5.

[5] Mogilner A, Oster G. Cell motility driven by actin polymerization. Biophys J. 1996 Dec;71(6):3030-45. doi: 10.1016/S0006-3495(96)79496-1.

[6] Altschuler SJ, Angenent SB, Wang Y, Wu LF. On the spontaneous emergence of cell polarity. Nature. 2008 Aug 14;454(7206):886-9. doi: 10.1038/nature07119.

[7] Nguyen TT, Park WS, Park BO, Kim CY, Oh Y, Kim JM, et al. PLEKHG3 enhances polarized cell migration by activating actin filaments at the cell front. Proc Natl Acad Sci U S A. 2016 Sep 6;113(36):10091-6. doi: 10.1073/pnas.1604720113.

[8] Neilson MP, Veltman DM, van Haastert PJ, Webb SD, Mackenzie JA, Insall RH. Chemotaxis: a feedback-based computational model robustly predicts multiple aspects of real cell behaviour. PLoS Biol. 2011 May;9(5):e1000618. doi: 10.1371/journal.pbio.1000618.

[9] Weiner OD. Rac activation: P-Rex1 - a convergence point for PIP(3) and Gbetagamma? Curr Biol. 2002 Jun 25;12(12):R429-31. doi: 10.1016/s0960-9822(02)00917-x.

[10] Inoue T, Meyer T. Synthetic activation of endogenous PI3K and Rac identifies an AND-gate switch for cell polarization and migration. PLoS One. 2008 Aug 27;3(8):e3068. doi: 10.1371/journal.pone.0003068.

[11] Byrne KM, Monsefi N, Dawson JC, Degasperi A, Bukowski-Wills JC, Volinsky N, et al. Bistability in the Rac1, PAK, and RhoA Signaling Network Drives Actin Cytoskeleton Dynamics and Cell Motility Switches. Cell Syst. 2016 Jan 27;2(1):38-48. doi: 10.1016/j.cels.2016.01.003.

[12] Guilluy C, Dubash AD, García-Mata R. Analysis of RhoA and Rho GEF activity in whole cells and the cell nucleus. Nat Protoc. 2011 Dec 1;6(12):2050-60. doi: 10.1038/nprot.2011.411.

[13] Burridge K, Wennerberg K. Rho and Rac take center stage. Cell. 2004 Jan 23;116(2):167-79. doi: 10.1016/s0092-8674(04)00003-0.

[14] Xu J, Wang F, Van Keymeulen A, Herzmark P, Straight A, Kelly K, et al. Divergent signals and cytoskeletal assemblies regulate self-organizing polarity in neutrophils. Cell. 2003 Jul 25;114(2):201-14. doi: 10.1016/s0092-8674(03)00555-5.

[15] van Leeuwen FN, Kain HE, Kammen RA, Michiels F, Kranenburg OW, Collard JG. The guanine nucleotide exchange factor Tiam1 affects neuronal morphology; opposing roles for the small GTPases Rac and Rho. J Cell Biol. 1997 Nov 3;139(3):797-807. doi: 10.1083/jcb.139.3.797.

[16] Wang Y, Ku CJ, Zhang ER, Artyukhin AB, Weiner OD, Wu LF, et al. Identifying network motifs that buffer front-to-back signaling in polarized neutrophils. Cell Rep. 2013 May 30;3(5):1607-16. doi: 10.1016/j.celrep.2013.04.009.

[17] Alberts B, Johnson A, Lewis J, Morgan D, Raff M, Roberts K, and Walter P, editors. Intracellular membrane traffic. New York: Garland Science; 2008.

[18] Mori Y, Jilkine A, Edelstein-Keshet L. Wave-pinning and cell polarity from a bistable reaction-diffusion system. Biophys J. 2008 May; 94(9):3684-3697. doi: 10.1529/biophysj.107.120824.

[19] Das S, Yin T, Yang Q, Zhang J, Wu YI, Yu J. Single-molecule tracking of small GTPase Rac1 uncovers spatial regulation of membrane translocation and mechanism for polarized signaling. Proc Natl Acad Sci U S A. 2015 Jan 20;112(3):E267-76. doi: 10.1073/pnas.1409667112.

[20] Zhang B, Zheng Y. Negative regulation of Rho family GTPases Cdc42 and Rac2 by homodimer formation. J Biol Chem. 1998 Oct 2;273(40):25728-33. doi: 10.1074/jbc.273.40.25728.

[21] Moissoglu K, Slepchenko BM, Meller N, Horwitz AF, Schwartz MA. In vivo dynamics of Rac-membrane interactions. Mol Biol Cell. 2006 Jun;17(6):2770-9. doi: 10.1091/mbc.e06-01-0005.

[22] Falkenberg CV, Loew LM. Computational analysis of Rho GTPase cycling. PLoS Comput Biol. 2013;9(1):e1002831. doi: 10.1371/journal.pcbi.1002831.

[23] Merchant B, Edelstein-Keshet L, Feng JJ. A Rho-GTPase based model explains spontaneous collective migration of neural crest cell clusters. Dev Biol. 2018 Dec 1;444 Suppl 1:S262-S273. doi: 10.1016/j.ydbio.2018.01.013.

[24] Takeuchi Y. Diffusion-mediated persistence in two-species competition Lotka-Volterra model. Math biosci. 1989;95(1):65–83. doi: 10.1016/0025-5564(89)90052-7.
